# Green synthesis of chitosan/erythritol/graphene oxide composites for simultaneous removal of some toxic species from simulated solution

**DOI:** 10.1007/s11356-022-23951-4

**Published:** 2022-11-09

**Authors:** Asmaa Sayed, Azza M. Mazrouaa, Manal G. Mohamed, Manar El-Sayed Abdel-Raouf

**Affiliations:** 1 Polymer Chemistry Department, National Center for Radiation Research and Technology, Egyptian Atomic Energy Authority, Cairo, Egypt; 2 Polymer Lab, Department of Petrochemicals, Egyptian Petroleum Research Institute, Nasr City, Cairo, Egypt; 3 Additives Lab, Department of Petroleum Application, Egyptian Petroleum Research Institute, Nasr City, Cairo, Egypt

**Keywords:** Chitosan, Erythritol, Graphene oxide, Water treatment, Mercury ions, Methylene blue dye, e-beam

## Abstract

In this study, chitosan (Ch) is adapted via green methodology including sonication induced crosslinking with different weight ratios of erythritol (Er) from (Ch-Er)_1_ to (Ch-Er)_4_. The products were casted in the form of thin films. The chemical modification was proved via FTIR spectroscopy. Then, the modified products were verified via an atomic force microscopy (AFM) investigation for their topography and surface properties. The data revealed that the optimized sample was (Ch-Er)_3_. This sample was further modified by different weight ratios of graphene oxide 0.1, 0.2, 0.4, and 0.8 wt./wt. (symbolized as (Ch-Er)_3_GO_1_, (Ch-Er)_3_GO_2_, (Ch-Er)_3_GO_4_, and (Ch-Er)_3_GO_8_ respectively). The prepared samples were investigated by different analytical tools. Then, the adjusted sample (Ch-Er)_3_GO_2_ was irradiated by electron beam (e-beam) at 10 and 20 kGy of irradiation doses to give samples (Ch-Er)_3_GO_2_R_10_ and (Ch-Er)_3_GO_2_R_20_, respectively. The AFM data of the irradiated samples showed that the pore size decreases, and surface roughness increases at higher energy e-beam due to the formation of more crosslinking points. The optimum samples of the prepared formulations were tested as sorbent materials for simultaneous elimination of methylene blue (MB) dye and mercury cation (Hg^2+^) from simulated solutions. The maximum removal of both MB dye and Hg^2+^ cation was achieved by (Ch-Er)_3_GO_2_R_10_ (186.23 mg g^−1^ and 205 mg g^−1^) respectively.

**Graphical abstract:**

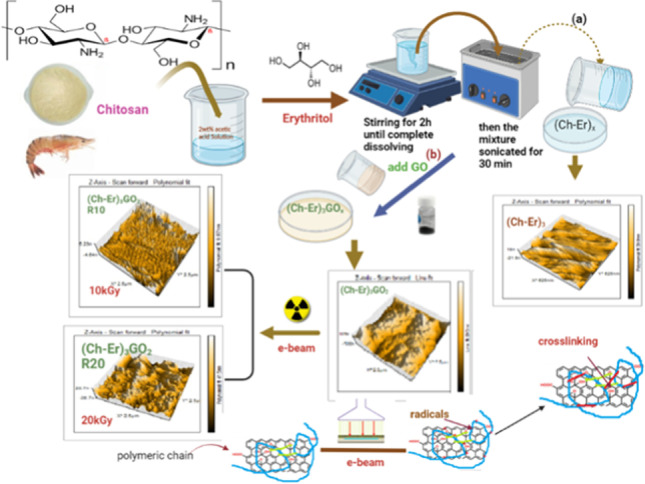

**Supplementary Information:**

The online version contains supplementary material available at 10.1007/s11356-022-23951-4.

## Introduction

Discharging the industrial effluents containing heavy metal cations and dyes into aquatic streams is one of the most critical wrongdoings that cause detrimental pollution for water and the terrestrial environments (Abdel-Raouf and Abdul-Raheim 2017, Al-Gorair et al. 2022, El-Kafrawy et al. 2017, Sayed et al. 2022b). The environment’s pollution by heavy metals induces different potent threats due to corruption of the food chain from base to top (M Abdul-Raheim et al. 2016, Zia et al. 2020). Mercury has been considered among the most hazardous heavy metal ions, including cadmium, arsenic, lead, and chromium (Mahmoud et al. 2022). The specific toxicity of mercury is mainly a consequence of its strong bioaccumulation and endurance in the food chain (Zhang et al. 2015a). One of the significant concerns of water treatment processes is reducing the excessive quantities of heavy metals to acceptable levels (Khozemy et al. 2020; Sayed et al. 2022a). Several protocols have been applied to remove mercury, including ion exchange, ultra-filtration, and adsorption. Indeed, adsorption has been verified to be the most practical and feasible solution (Ali et al. 2022). Green sorbents have been introduced as effective alternatives to petroleum-based sorbents (Al-Qudah et al. 2022; Teng et al. 2021). Chitosan is among the most abundant high-functional natural polymers (Ghobashy et al. 2021). It can be adapted to improve its properties, such as biodegradability, sorption capacity, and sustainability (Elbarbary and Ghobashy 2017, Wang et al. 2016). The occurrence of amino groups in the chitosan backbone induces high catalytic activity, metal chelating ability, and biological efficiency (Mahmoud et al. 2020). Several protocols were applied for the modification of chitosan. In this regard, a chitosan ethylene glycol hydrogel (ChEGH) was created through repeated thawing and freezing. The prepared hydrogel was employed as a sorbent for organic pollutants. Experimental findings demonstrated that the ionic hydrogen bond between the (NH^+^) group of the chitosan backbone and carbonyl groups (COO^−^) of perfluorooctanoic acid (PFOA) was the principal elimination mechanism (Long et al. 2019).

Conversely, polyols are strong hydrophilic compounds that can bind with water molecules through hydrogen bonds, resulting in moisture absorption. Chitosan plasticizers include polyethylene glycol, sorbitol, and glycerol (Lavorgna et al. 2010). Specifically, glycerol can upsurge the biopolymer chain mobility by reducing the intermolecular forces (Rivero et al. 2016). Films of chitosan were plasticized with the glycerol and AlCl_3_.6H_2_O complex to increase their water resistance (Dong et al. 2019). On the other hand, carbon complexes were proved as possible sorbents for many pollutants. Therefore, graphene oxide was selected—an effective candidate of the carbon family—due to its exceptional structure (2-D, one-atom-thick structure), superior mechanical qualities, and total surface area for chitosan modification (Sayed et al. 2022c). Graphene oxide (GO) has been widely employed to eliminate metal ions, dyes, and other contaminants (Gad and Nasef 2021, Kameli and Mehrizad 2019, Mehrizad and Gharbani 2014). The chitosan-GO grafted materials were nominated due to functional group variation (OH, H, CO, and NH_2_) in the composite matrix, which can interact with many pollutants in different ways (Kumar and Jiang 2016, Zhao et al. 2016).

This work’s primary purpose is to prepare green adsorbents based on modified chitosan to eliminate methylene blue dye and mercury cations as model pollutants from simulated aqueous solutions. The hydrogels were prepared by sonication as an effective, clean, and energy-saving method for radical initiation and cross-linking. The thermal properties, crystallinity, and surface topography of the prepared materials are also investigated thoroughly. The effect of time and temperature on the removal performance was also studied. An atomic force microscopy (AFM) investigation was performed to correlate the surface features with the maximum removal capacity. Moreover, the effect of electron beams on the surface features was studied, and the hydrogel’s efficiency before and after exposure to different irradiation doses was compared by the AFM and the batch removal experiments.

## Materials and methods

### Materials

Chitosan (Ch) ˃ 75% degree of deacetylation with Mw. of 110 Da. was purchased from Sigma-Aldrich company. Meso-erythritol C_4_H_10_O_4_, 99% was obtained from Alfa Aesar company. Acetic acid was purchased from Al-Nasr chemicals (Egypt), as a 99% grade reagent. Graphene oxide (GO), 1% aqueous dispersion equivalent to 10 mg/mL was obtained from William Blythe Excellence in Chemistry, UK. Mercuric chloride salt and methylene blue dye were obtained from Sigma-Aldrich Co.

### Methods

#### Preparation of chitosan/meso-erythritol/ graphene oxide film

**Scheme 1:**
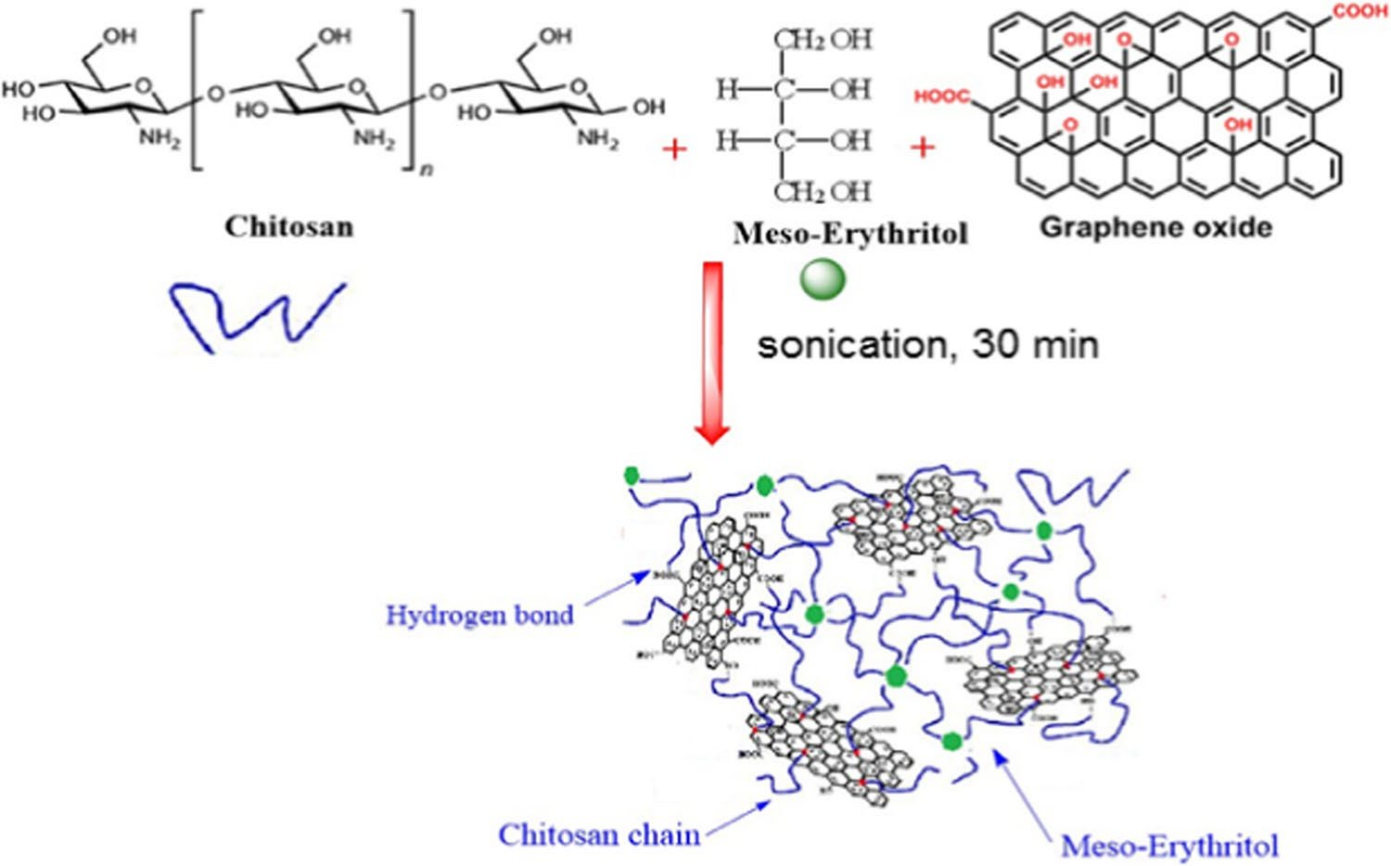
Schematic representation of synthetic procedure of chitosan/erythritol/graphene oxide hydrogels

The green chitosan (Ch) reinforced films were synthesized by the facile green sono-chemical process. Briefly, 10 g of Ch was dissolved in 100 mL of 2% (v/v) CH_3_COOH aqueous solution and sonicated with a tip sonicator (JY92-II, 6 mm stepped microtip, Xinzhi Inc., Hangzhou, China). The solution was filtered to remove undissolved impurities using vacuum filter. After filtration, the solution was returned back to the sonicator, then meso-erythritol (Er) was added and the solution and sonicated again to achieve complete dispersion. The formulations were coded according to the percentage of erythritol as (Ch-Er)_x_ where x is different ratio 10 wt %, 20 wt %, 30 wt %, and 40 wt % Er to Ch (Ch-Er)_1_, Ch-Er)_2_, (Ch-Er)_3_, and (Ch-Er)_4_, respectively, Table 1. Then, GO was added stepwise to the (Ch-Er)_3_ solution at the desired weight ratios with vigorous stirring, and the mixture was further sonicated for 2 h. The composition ratios of (Ch-Er)_3_/GO nanocomposites were denoted as (Ch-Er)_3_GO_y_, where the y is 0.1, 0.2, 0.4, and 0.8 mL of graphene oxide respectively (Table 1). The films were vacuum-dried at 60 °C for 24 h. Then, the selected (Ch-Er)_3_GO_2_ sample was subjected to an optimized dose of e-beam irradiation 10 and 20 kGy, and these are coded as (Ch-Er)_3_GO_2_R10 and (Ch-Er)_3_GO_2_R20 respectively. It was found that (Ch-Er)_3_GO_2_ became brittle and easily cracked when exposed to a dose of more than 20 kGy. The diagram illustrating the synthetic procedure of (chitosan-erythritol)/graphene oxide hydrogels is shown in Scheme 1.

**Table 1:** Codes of (Ch-Er)_x_ and (Ch-Er)_x_GO_y_ nanocomposites

Code	Ch (mL)	Er (g)	GO (mL)
(Ch-Er)_1_	10	0.1	0
(Ch-Er)_2_	10	0.2	0
(Ch-Er)_3_	10	0.3	0
(Ch-Er)_4_	10	0.4	0
(Ch-Er)_3_GO_1_	10	0.3	0.1
(Ch-Er)_3_GO_2_	10	0.3	0.2
(Ch-Er)_3_GO_4_	10	0.3	0.4
(Ch-Er)_3_GO_8_	10	0.3	0.8

## Electron beam irradiation

(Ch-Er)_3_GO_2_ samples were irradiated utilizing a 3-MeV e-beam linear accelerator placed at the National Center for Radiation Research and Technology (NCRRT), Egyptian Atomic Energy Authority (EAEA), Cairo, Egypt (operating parameters: conveyer speed of 16 m/min (50 HZ), beam current of 30 mA, power of 90 kW, and variable scan width up to 90 cm).

## Characterization

A Perkin-Elmer 1650 FTIR spectrophotometer was used to conduct an FTIR analysis. A WA-XRD was used to analyze the crystal structure of the materials. The peaks were recorded according to the 2θ range with a time of 0.7000 s and a step size of 0.02˚A. The surface topography and roughness were verified via atomic force microscopy (AFM), Flexaxiom Nanosurf C3000, Nanosurf, located at the Egyptian Petroleum Research Institute, in a dynamic mode using a cantilever NCLR. The thermal characteristics were determined to examine the structural modifications caused by thermal treatment on the designed materials. On an SDTQ-600 (TA-UAS) thermo balance instrument, thermal characteristics were monitored by heating 15 mg of the sample to 600 °C at a heating rate of 10 °C/min at a nitrogen flow rate of 100 mL/min. The dye concentration was monitored via the UV spectrophotometer model Jasco V-750, while an AA-670 Shimadzu atomic absorption spectrometer (AAS) was used to examine the nanocomposite’s metal absorption (Japan).

### Batch adsorption experiments

Sorption experiments were conducted for the optimization of the prepared formulations. In this regard, batch adsorption experiments were performed via the tea bag method to compare the performance of chitosan green composites versus methylene blue dye and Hg^2+^. The polymer (0.05 g) was put in a tea bag and placed in a simulated aqueous solution containing 50 ppm of the tested pollutant at room temperature (25 °C). Then, 1 mL of the solution is drawn at different time intervals to monitor the removal rate and percentage until 6 h. An empty tea bag was used as a blank at the same application condition of each experiment. The removal percentage (%) was calculated by the following equation (Boudrahem et al. 2009; Zhao et al. 2016):

1 $$Removal\left(\%\right)=\frac{\left({C}_{o}-{C}_{e}\right)}{{C}_{o}}\times 100$$

where *C*_0_ (mg L^−1^) is the initial pollutant concentration; *C*_*e*_ (mg L^-1^) is the equilibrium concentration of the adsorbate.

The adsorption of toxic (Hg^2+^) ions and MB was demarcated with the adsorption kinetics, and the pseudo-1^st^-order and pseudo-2^nd^-order kinetics in non-linear forms, Eqs. (2) and (3), respectively, were applied to adsorption process (Al-Gorair et al. 2022) as follows:

2 $${q}_{t}={q}_{e}\left(1-{e}^{-{k}_{1}t}\right)$$

3 $${q}_{t}=\frac{{k}_{2}{q}_{e}^{2}t}{1+{k}_{2}{q}_{e}t}$$

where *q*_*t*_ and *q*_*e*_ are the concentrations of MB or toxic (Hg^2+^) ions adsorbed at time *t* and equilibrium *e*, respectively; *k*_1_ and *k*_2_ are the pseudo-1^st^-order and pseudo-2^nd^-order rate constants for the adsorption, respectively. In a multistep process, the adsorbate may migrate from the solution phase to the surface of the adsorbent. Surface diffusion, film or external diffusion, adsorption on the pore surface, and pore diffusion may all be involved in the process (Kavand et al. 2017). The process of adsorption was evaluated by three isotherm models, Langmuir (Eq. 4), Freundlich (Eq. 5), and Redlich-Peterson (Eq. 6). The calculations are made according to the following non-linear mathematical relations (Zhang et al. 2015b):

4 $$\begin{array}{c}\begin{array}{cc}\mathrm{Langmuir}\;\mathrm{model}:&q_e=\frac{q_mK_LC_e}{1+K_LC_e}\end{array}\end{array}$$

where *K*_*L*_ (mg/L) and *q*_*m*_ (mg/g) are the Langmuir constants for adsorption energy and sorption power, respectively. Hence, *q*_*e*_ (mg/g) refers to the equilibrium adsorption capacity, and *C*_*e*_ (mg/L) is defined as the equilibrium concentration.

5 $$\mathrm{Freundlich\;model}: {q}_{e}={K}_{F}{C}_{e}^{1/n}$$

where the Freundlich constant *K*_*f*_ (mg/L) indicates the sorption capacity and the sorption intensity is expressed by *n*, which represents the heterogeneity function.

6 $$\mathrm{Redlich}-\mathrm{Peterson\;model}: {q}_{e}=\frac{{K}_{RP}{C}_{e}}{1+{a}_{R}{C}_{e}^{g}}$$

where, ${K}_{RP}, {a}_{R},\mathrm{and} g$ are Redlich-Peterson’s three parameters, and $"g"$ lies between 0 and 1. In order to study the effect of temperature and the removal kinetics, the former procedure was repeated at three temperatures, 35, 45, and 55 °C.

## Results and discussion

### Fourier transform infrared spectroscopy

The FTIR spectrum of chitosan depicts significant peaks at 1061 and 1033 cm^−1^ (C–O–C stretching) and 2890 cm^−1^ (CH_2_ stretching). An observed band within the range of 3295–3365 cm^−1^ was assigned to N–H and O–H stretching and the intramolecular hydrogen bonds. Two overlapped bands at 1590 and 1640 cm^−1^ correspond to the N–H bending of the amide group (Mahmoud et al. 2020). The band at around 1375 cm^−1^ is assigned for CH_2_ bending. All bands are detected in the spectra of chitosan samples described by various studies (Duraisamy et al. 2022; Sethi et al. 2022). The FTIR spectrum of dry erythritol shows the common peaks of an alcoholic compound. The strong peak at 3251 cm^−1^ is assigned for stretching vibration of free O–H. Both C–H symmetric and asymmetric stretching are assigned to the two medium peaks at 2968 and 2957 cm^−1^. The absorption band at 1181 cm^−1^ is attributed to the asymmetric stretching of the C–O bond. The significant peaks at 1560 and 1467 cm^−1^ are assigned for CH_2_ bending and CH symmetrical vibrations, respectively, as shown in Figure S2. The peak ascribed to amine functional groups changed dramatically after further treatment with GO. The peak at ~ 1647 cm^-1^ vanished, and the peak at 1520 cm^-1^ was drastically lowered. Also, the C = C bond that is often detected in the range ~ 1631 cm^-1^ of graphene spectra (Corazzari et al. 2015) was stifled. These findings propose the interaction of –NH_2_ groups of chitosan and C–C bonds of GO that may lead to the development of new C–N or C–H bonds. As demonstrated in Figure S2, the peak at ~ 1020 cm^-1^ allocated to the C–N stretch (Huang et al. 2020) is thus attributable to the interaction between the C–C bonds of graphene and the amine functional groups of chitosan. The characteristic peaks of GO appear in the region of 520–720 cm^-1^, and the most important peaks are given in (Supplementary data Figure S2 and Table S1).

#### AFM study of chitosan-erythritol hydrogels

AFM is an effective and consistent satisfactory practice for confirming the chemical modification of different materials. Four chitosan-erythritol ratios were applied to investigate erythritol’s effect on creating a porous structure with chitosan (Supplementary data Figure S3 and Table S2). The data were filtered by the parabola fit to go through the deep details. The image of chitosan shows a sinuous surface with low height (15.9 nm) and the least average roughness. The zigzags of the chitosan surface may be attributed to the intramolecular hydrogen bonding of primary amine groups and N-acetyl glucose amine units. Upon modification, the change in both the topography and the roughness measurement reflects the current amendments.

For instance, the height increased with the erythritol percentage, i.e., the maximum height was attained at the highest erythritol content. The height reflects the pore volume. For optimum sorption, moderate pores are preferred to absorb and retain the pollutant. If the pores are very narrow, the sorption will not be effective. Also, in the case of very wide pores, the sorbent pollutants can escape from the matrix. Then, according to the height measurement, it can be expected that (Ch-Er)_3_ would give maximum removal; the elimination experiments proved this prediction. These findings agree with our previous work (Keshawy et al. 2022).

#### AFM study of chitosan-erythritol/GO hydrogels

**Fig. 1:**
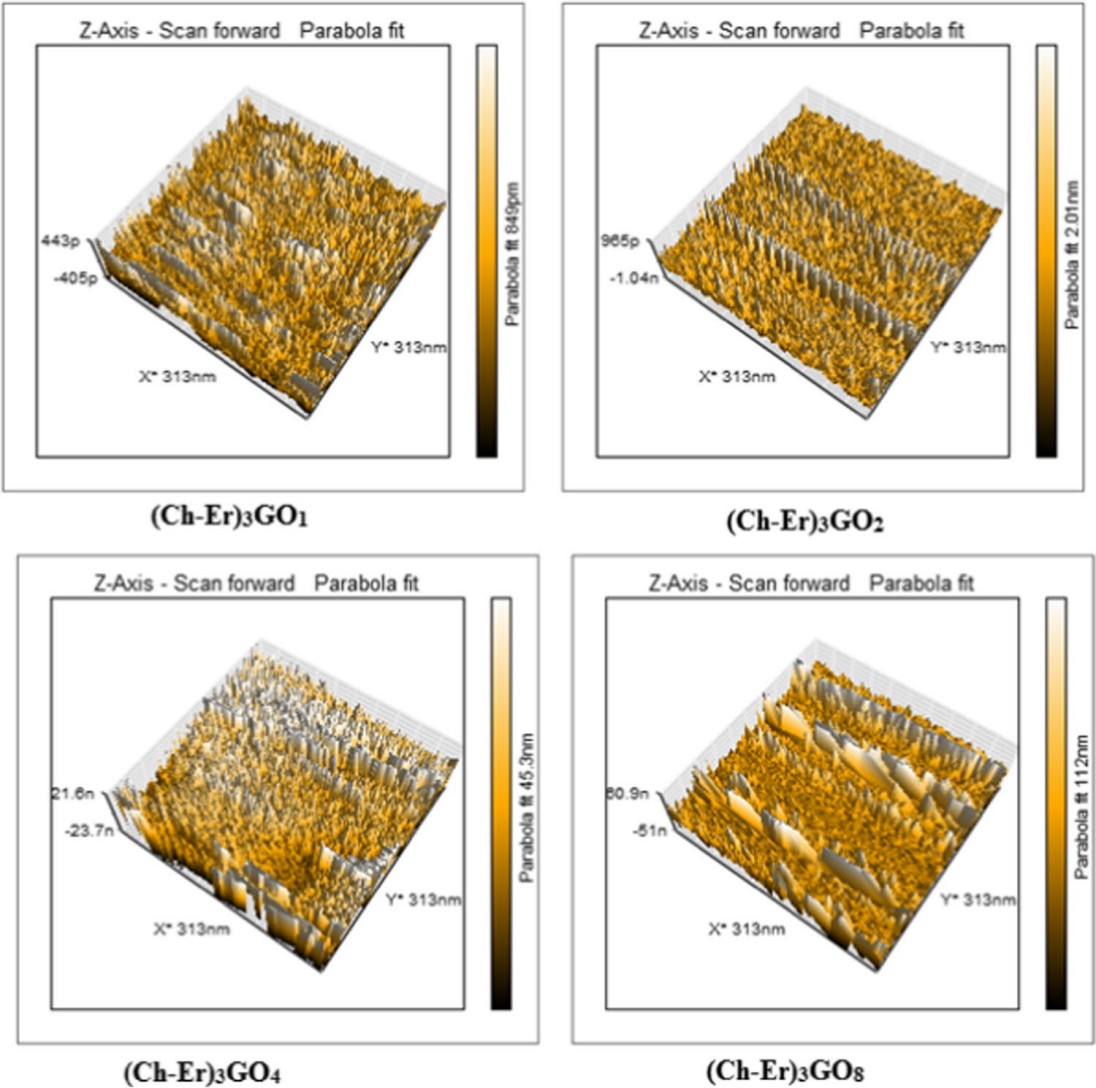
The AFM images of (Ch-Er)_3_GO_1_, (Ch-Er)_3_GO_2_, (Ch-Er)_3_GO_4_, and (Ch-Er)_3_GO_8_

**Fig. 2:**
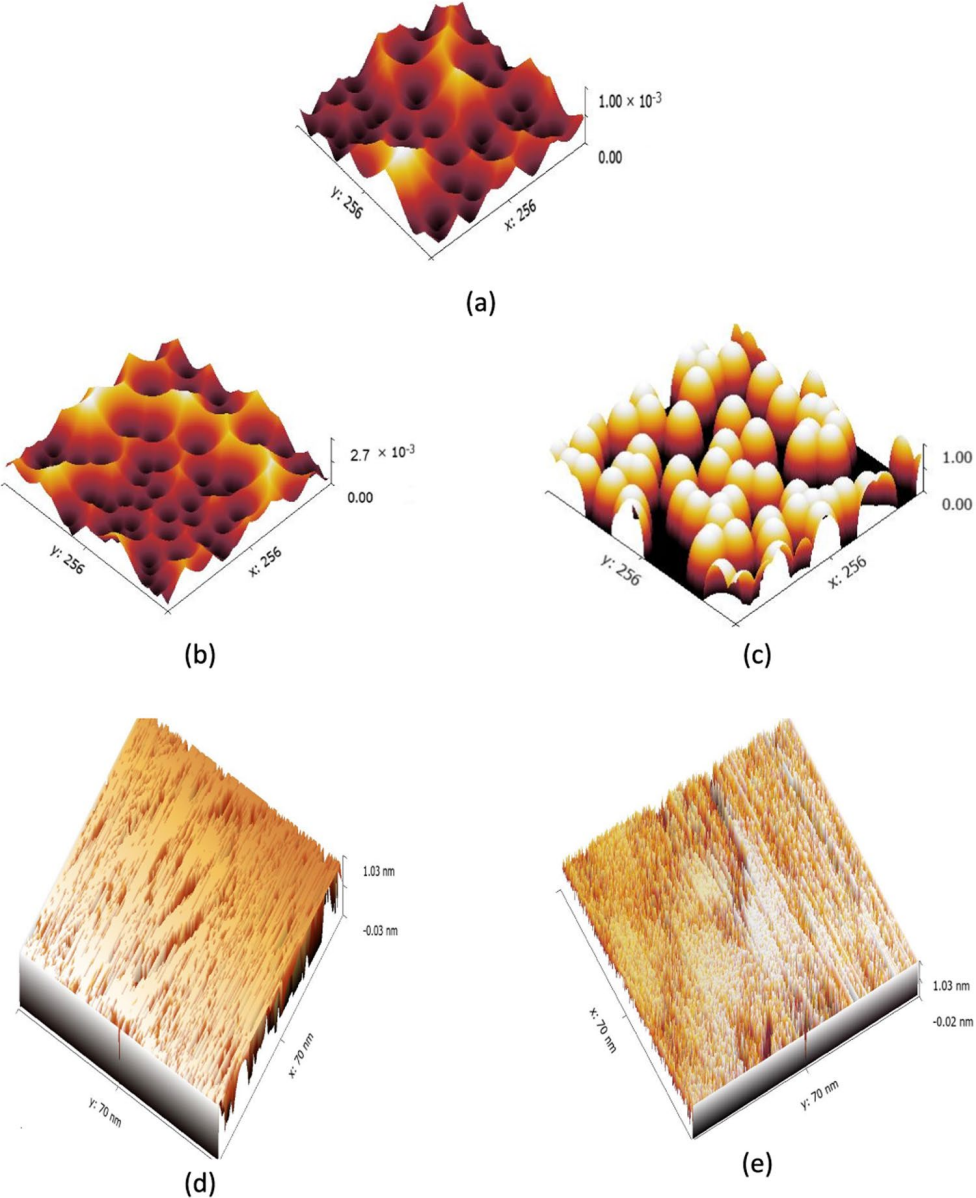
Porous structure of (Ch-Er)_3_ (**a**); (Ch-Er)_3_GO_2_ (**b**); GO particles in (Ch-Er)_3_GO_2_ (**c**); after analyzed via Gwydion software (Ch-Er)_3_ (**d**), and (Ch-Er)_3_GO_2_ (**e**)

Based on the previous AFM data, (Ch-Er)_3_ was further modified with four weight ratios of graphene oxide incorporated into the hydrogel matrix. They are coded according to the ratio of GO as (Ch-Er)_3_GO_1_, (Ch-Er)_3_GO_2_, (Ch-Er)_3_GO_4_, and (Ch-Er)_3_GO_8._ The AFM images of these composites are given in Fig. 1, and the AFM outcomes are provided in Table 2. Moreover, the images of (Ch-Er)_3_ and (Ch-Er)_3_GO_2_ are further analyzed to illustrate both the porous structure and the graphene oxide, Fig. 2.

**Table 2:** AFM data of (Ch-Er)_3_GO_1_, (Ch-Er)_3_GO_2_, (Ch-Er)_3_GO_4_, and (Ch-Er)_3_GO_8_

No	Sample	Height	Scale	Data analysis	R_a_ _(average area roughness)_
	(Ch-Er)_3_GO_1_	0.85 nm	313 × 313 nm	Parabola fit	23.23 nm
	(Ch-Er)_3_GO_2_	2.01 nm	313 × 313 nm	Parabola fit	27.41 nm
	(Ch-Er)_3_GO_4_	45.3 nm	313 × 313 nm	Parabola fit	31.71 nm
	(Ch-Er)_3_GO_8_	112 nm	313 × 313 nm	Parabola fit	34.54 nm

With the careful inspection of the AFM images and the derived data, it can be observed that surface roughness and height both increased by an increment of the amount of graphene oxide incorporated into the hydrogel matrix. As the height reflects the pore volume, the highest height is not favored for adsorption due to detachment of the adsorbed species. Therefore, the optimum samples are expected to be (Ch-Er)_3_GO_1_ or (Ch-Er)_3_GO_2_ according to height measurements (Khan et al. 2021). Moreover, the graphene oxide particles are deposited clearly on the surface in the form of clustered masses, parallel to the added graphene oxide concentration.

For better comparison, two AFM images for (Ch-Er)_3_ and (Ch-Er)_3_GO_2_ were analyzed via Gwyddion software. The brush structure of (Ch-Er)_3_GO_2_ due to the incorporation of GO can be distinguished in Fig. 2d, e.

#### AFM study of (Ch-Er)_3_GO_2_R10 and (Ch-Er)_3_GO_2_R10 irradiated by electron beams

**Fig. 3:**
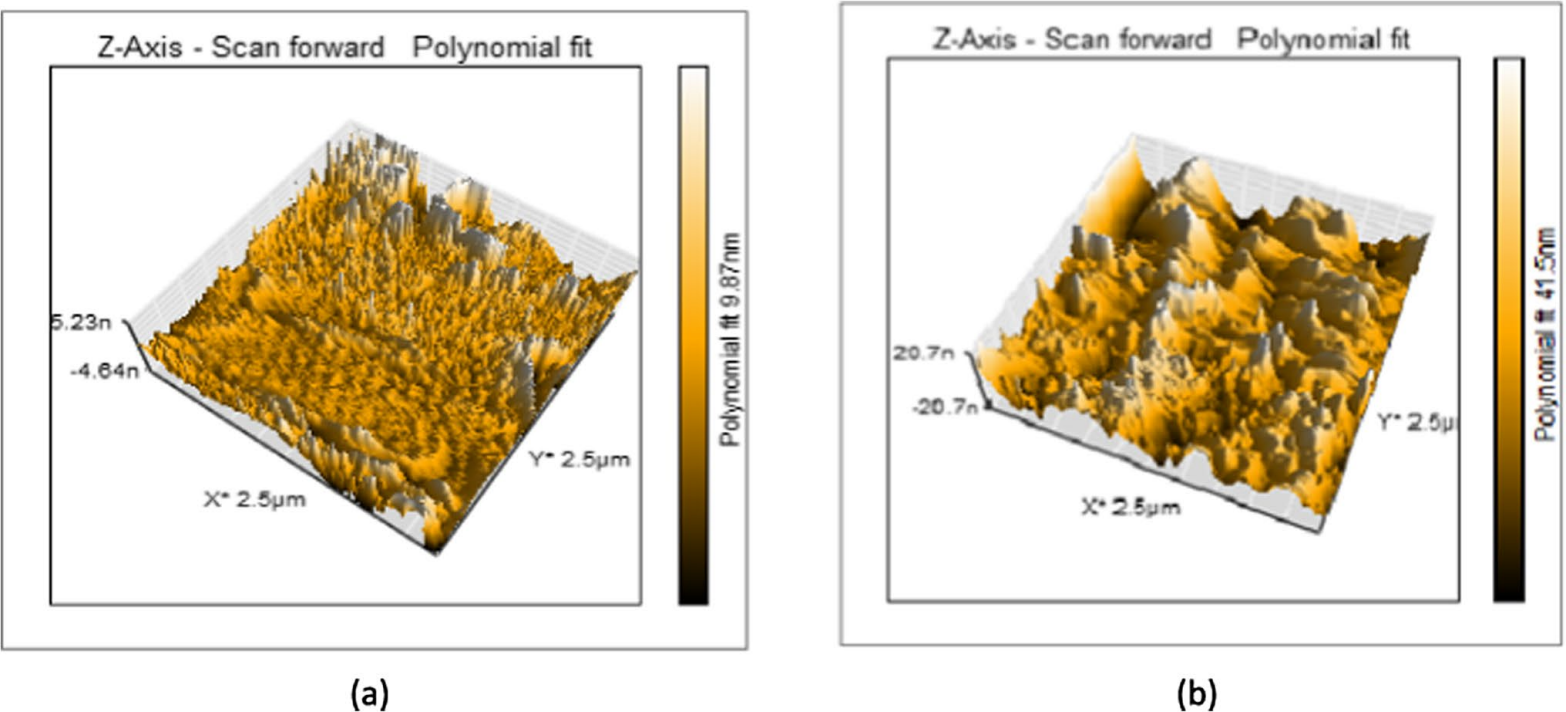
AFM images of irradiated (Ch-Er)_3_GO_2_R_10_ (**a**) and (Ch-Er)_3_GO_2_R_20_ (**b**)

An AFM investigation was also conducted to verify the effect of electron beam irradiation on the samples’ surface features, which implied better performance, i.e., (Ch-Er)_3_GO_2_R_10_ and (Ch-Er)_3_GO_2_R_20_. The images are provided in Fig. 3. There are several apparent clusters scattered randomly, which implies more cross-linking points. The height is less than their counterparts due to the irregular and disturbed distribution of cross-linking points within the hydrogel matrix. This results in reduced pore volume, random pore distribution, and poor efficiency. This observation proves that the irradiation cross-linking must be well controlled to regulate the number of pores and their volume (Hafezi Moghaddam et al. 2020). Our data agrees with Khan et al. (Khan et al. 2021) and Huang et al. (Huang et al. 2012).

### Scanning electron microscopy (SEM)

SEM imaging provides direct evidence concerning the interfacial interactions of pure Ch, (Ch-Er)_3_, and (Ch-Er)_3_GO_2_ composite films (Supplementary data Figure S4). The usually smooth shape of the Ch surface may be attributed to its proclivity to self-aggregate in solution (Bardajee and Hooshyar 2013, Jiang et al. 2016, Pandey and Mishra 2011). For the chain entangling and physical cross-linking of the (Ch-Er)_3_, the inner structure of (Ch-Er)_3_GO_2_ is much denser than those of Ch and (Ch-Er)_3_, which predicts the enhancement of the hydrogel. The apparent increase in density of the blended films in contrast to the pure chitosan film reflects the strong interaction between chitosan/erythritol and graphene oxide. However, as the blended GO is wrapped in or covered by a network of (Ch-Er)_3_ layer, there is hardly any totally exfoliated graphene oxide, indicating a strong bond between the (Ch-Er)_3_ and graphene oxide.

### X-ray diffraction (XRD)

**Fig. 4:**
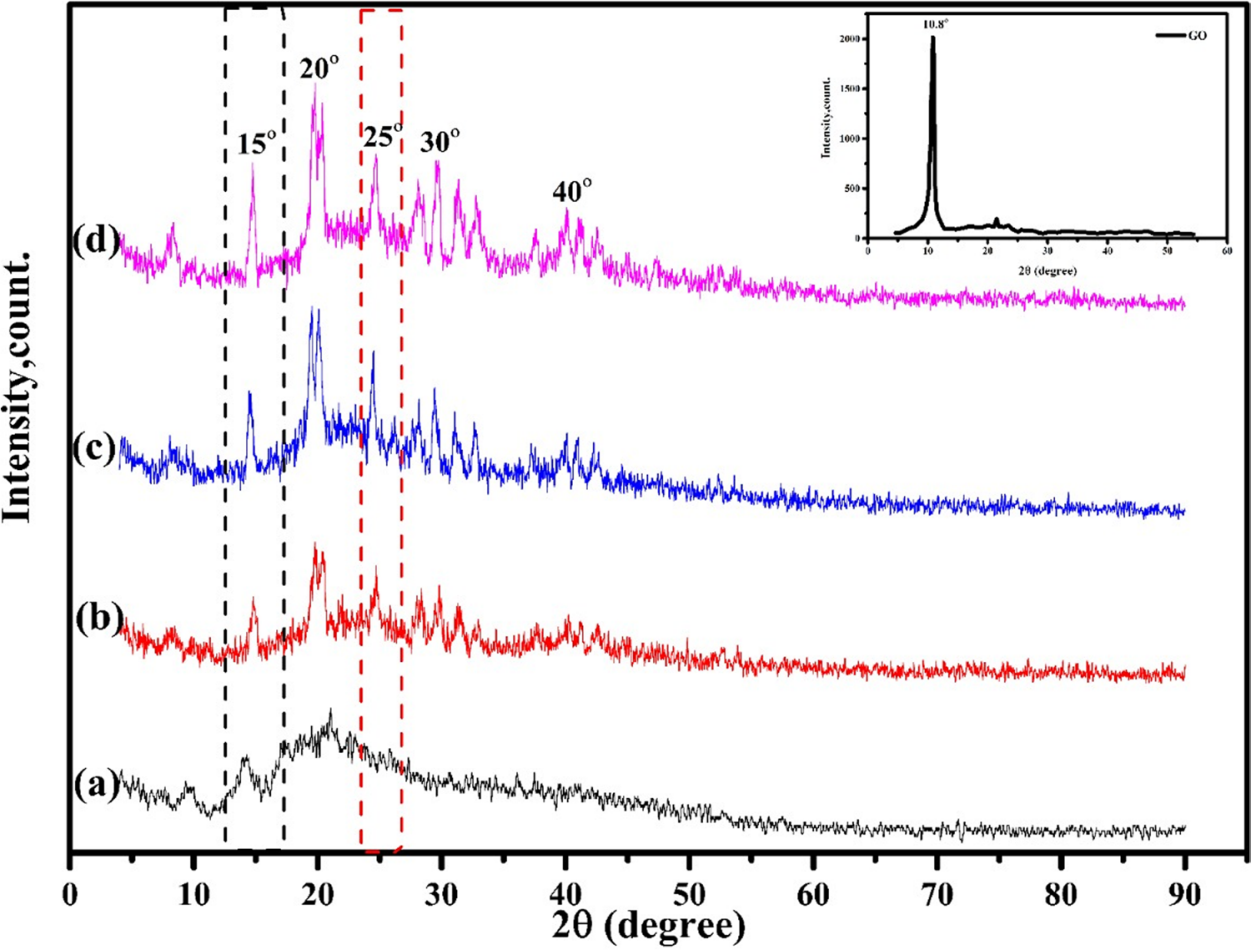
XRD patterns of (**a**) GO, (**b**) (Ch-Er)_3_GO_1_, (**c**) (Ch-Er)_3_GO_2_, (**d**) (Ch-Er)_3_GO_2_R_10_

The XRD patterns of GO, (Ch-Er)_3_GO_1_, (Ch-Er)_3_GO_2_, and (Ch-Er)_3_GO_2_R_10_ are shown in Fig. 4. All the prepared films exhibited two sharp peaks at 2θ = 15.0° and 20° due to the general amorphous state of the chitosan films. The diffraction angles of the composite films (Ch-Er)_3_GO_2_ are almost identical to those of pure Ch, and the graphene oxide diffraction peaks have not been identified owing to their overlap. This indicates that the exfoliation of GO occurred due to the high dispersion in the network of (Ch-Er)_3_ (Lim et al. 2012; Subedi et al. 2019). In addition, after exposure of (Ch-Er)_3_GO_2_ to 10 kGy, the intensity of GO peaks increased, indicating that the distribution of GO in the polymeric matrix became more homogenous.

### Thermal stability analysis (TGA)

**Fig. 5:**
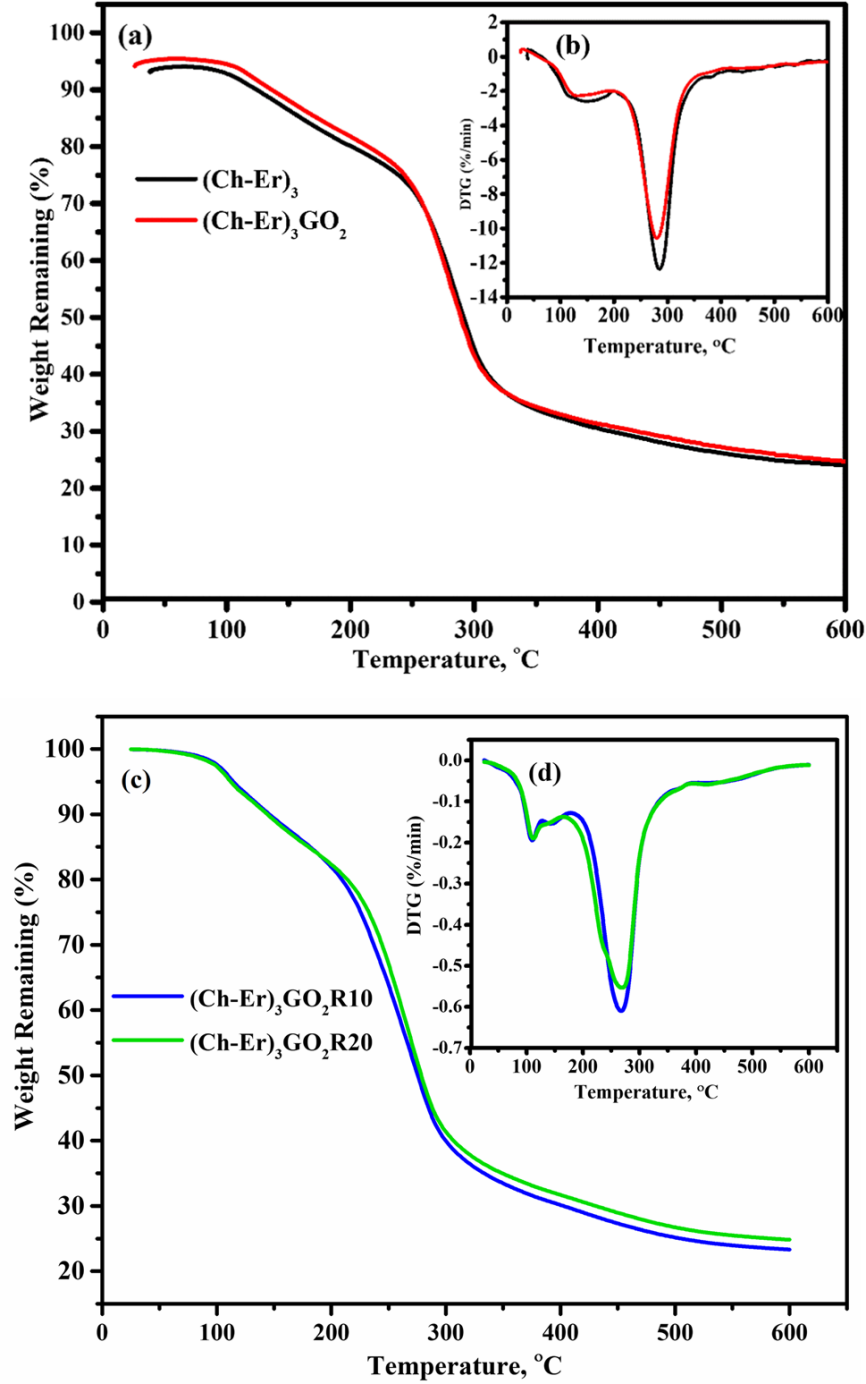
TGA and DTA thermograms of: (Ch-Er)_3_ and (Ch-Er)_3_GO_2_ (**a** and **b**), (Ch-Er)_3_GO_2_R10 and (Ch-Er)_3_GO_2_R20 (**c** and **d**)

Graphene and other carbon elements create a reinforcing effect that reduces segmental chain mobility, resulting in improved thermal and tensile properties of composite films (Abolhassani et al. 2017). Due to the increased degree of limitation in the mobility of the polymeric chitosan chains, this may explain how the bigger GO particles contributed to the better thermal characteristics of the chitosan films (Bao et al. 2011). (Ch-Er)_3_ and (Ch-Er)_3_GO_2_ thermal behavior was evaluated by thermogravimetric analysis (Fig. 5a), and their DTG graphs are given in Fig. 5b. The thermal decomposition patterns of the irradiated compounds are given in Fig. 9c, d for TGA and DTG, respectively. The degradation patterns of (Ch-Er)_3_ displayed two degradation stages. The first one lies in the region of 0–250 °C, where the weight slightly decreased due to the decomposition of carboxylic groups and the release of CO_2_ gas (Yang et al. 2010). The second mass loss of Ch is observed at about 300 °C due to the cross-linked structure’s decomposition (Alferez Luna et al. 2021; Ilyas et al. 2022).

On the other hand, the TGA curve of (Ch-Er)_3_GO_2_ composite shows two degradation steps. The first step starts from 38–259 °C with weight loss of 14 wt.%, due to the evaporation of H_2_O and moisture, while the second step degradation appears in the region 210–786 °C with weight loss of 65.5%. In addition, the chemical bond between chitosan chains and GO sheets in GO-chitosan composite films may constrain the conformities of polymer molecules to a greater extent than intermolecular forces, resulting in a larger Tg for the composite. This stability is probably due to the increased hydrogen bonding interaction between erythritol, chitosan, and GO (Bakshi et al. 2020; Samuel et al. 2018).

On the other hand, the TGA thermograms of the irradiated samples show slightly higher thermal stability of (Ch-Er)_3_GO_2_R_20_ than (Ch-Er)_3_GO_2_R_10_ (Fig. 5c). This may be attributed to the increased cross-linking due to the higher dose of e-beam. Thus, the DTG curve displays a lower rate of degradation (Fig. 5d).

### Adsorption process

#### Effect of graphene oxide content on removal performance

**Fig. 6:**
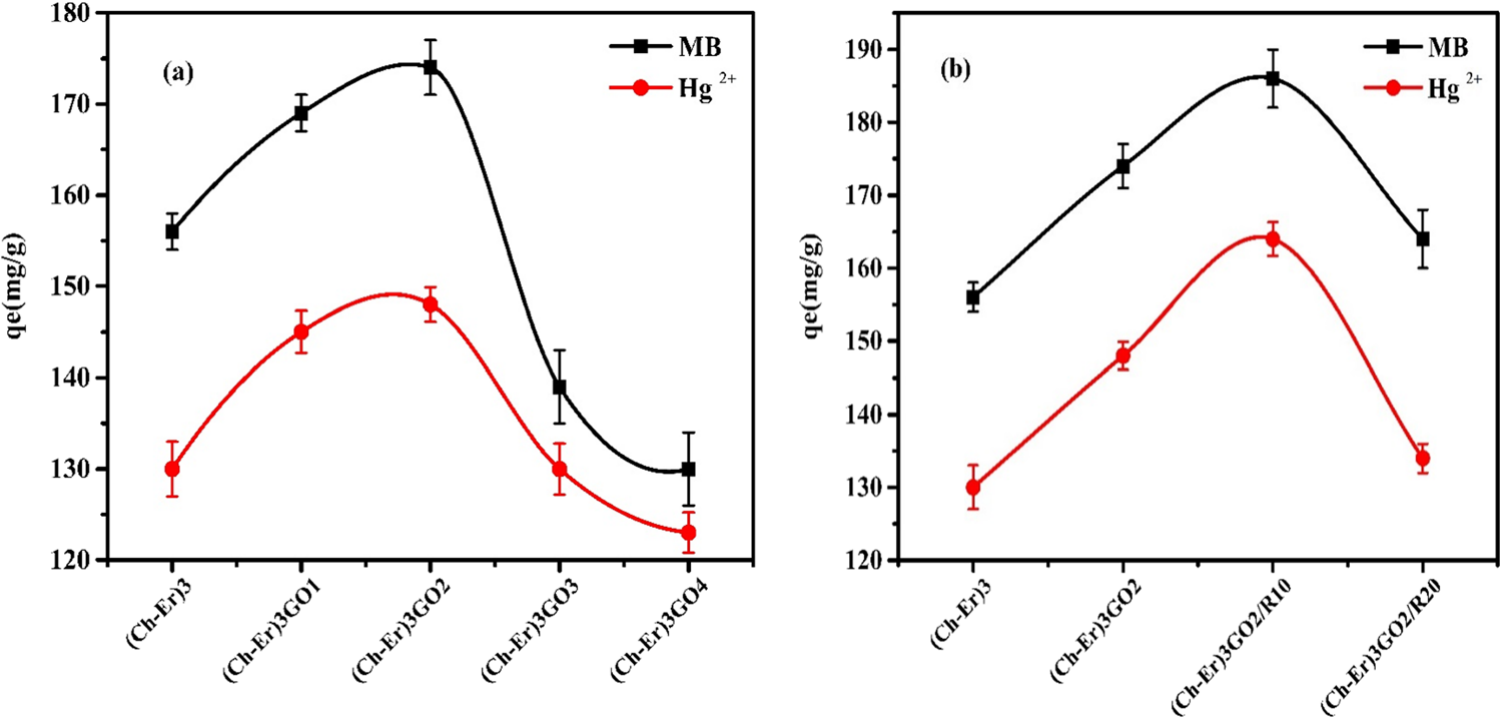
Effect of GO content on removal capacity (*qe* mg/g) of MB and Hg^2+^ (**a**) and comparison of the removal capacity (*qe* mg/g) between (Ch-Er)_3_, (Ch-Er)_3_GO_2_, (Ch-Er)_3_GO_2_R_10_, and (Ch-Er)_3_GO_2_R_20_ (**b**); initial conc. 50 ppm; adsorbate weight 0.1 g at room temperature

The removal performance of the chitosan/erythritol/graphene oxide composites towards MB dye and Hg^2+^ ions is illustrated in Fig. 6a. It was found that a moderate ratio of graphene oxide is suitable for obtaining maximum removal efficiency, i.e., at 2% GO. Above this ratio, the removal performance versus either methylene blue dye or mercury cations is reduced remarkably. This may be explained by SEM that the graphene oxide is distributed homogenously at low concentrations (Supplementary data Figure S4). On the contrary, at high concentrations, the graphene oxide particles are clustered and unequally distributed within the matrix, blocking many active sites, which finally hinders the uptake process. Other works also suggested this conclusion (Subedi et al. 2019; Teng et al. 2021; Zhang et al. 2016).

However, upon comparing the removal performance of (Ch-Er)_3_/GO_x_ composites towards the claimed pollutants, it can be seen that all the chitosan-based sorbents displayed higher uptake towards methylene blue dye than mercury cations. This observation may infer the nature of the removal process by this type of sorbent, which is dependent mainly on the interaction between the surface’s active sites and the adsorbed species. This also may be because the erythritol is a small spacer resulting in very narrow pores and the sorbents prefer superficial adsorption over interior adsorption.

In order to investigate the effect of electron beam irradiation on the removal process, (Ch-Er)_3_GO_2_ was exposed to two irradiation doses (10 and 20 kGy). The removal performance of MB and Hg^2+^ onto (Ch-Er)_3_, (Ch-Er)_3_GO_2_, (Ch-Er)_3_GO_2_R_10_, and (Ch-Er)_3_GO_2_R_20_ is illustrated in Fig. 6b. It is observed that the removal performance was enhanced at a low irradiation dose due to the addition of more cross-linking points. However, when the irradiation dose increased, the surface morphology changed, and several cross-linked clusters were distributed irregularly, leading to inhibition of the adsorption process. Our finding runs parallel to Calina et al. (Călina et al. 2020). It is demonstrated that (Ch-Er)_3_GO_2_R_10_ achieved maximum removal due to the proper modification of the surface by an adjusted dose of irradiation.

#### Time-dependent removal 

Time-dependent removal performance for the prepared (Ch-Er) _x_ (Supplementary data Figures S5a and S5b) and of (Ch-Er)_3_, (Ch-Er)_3_GO_2_, and (Ch-Er)_3_GO_2_R_10_ (Supplementary data Figures S6a and S6b) for methylene blue dye and mercury cations, respectively, has been studied. The data revealed that the rate and percentage of removal increased rapidly over time and that the most effective adsorbent is (Ch-Er)_3_. This finding is consistent with the AFM investigation outcomes and the prepared hydrogels’ topographical features. This behavior may be attributed to the filling of voids and active sites of the adsorbent material with adsorbate over time until reaching saturation (Huang et al. 2021; Khan et al. 2021).

##### Kinetic studies

**Fig. 7:**
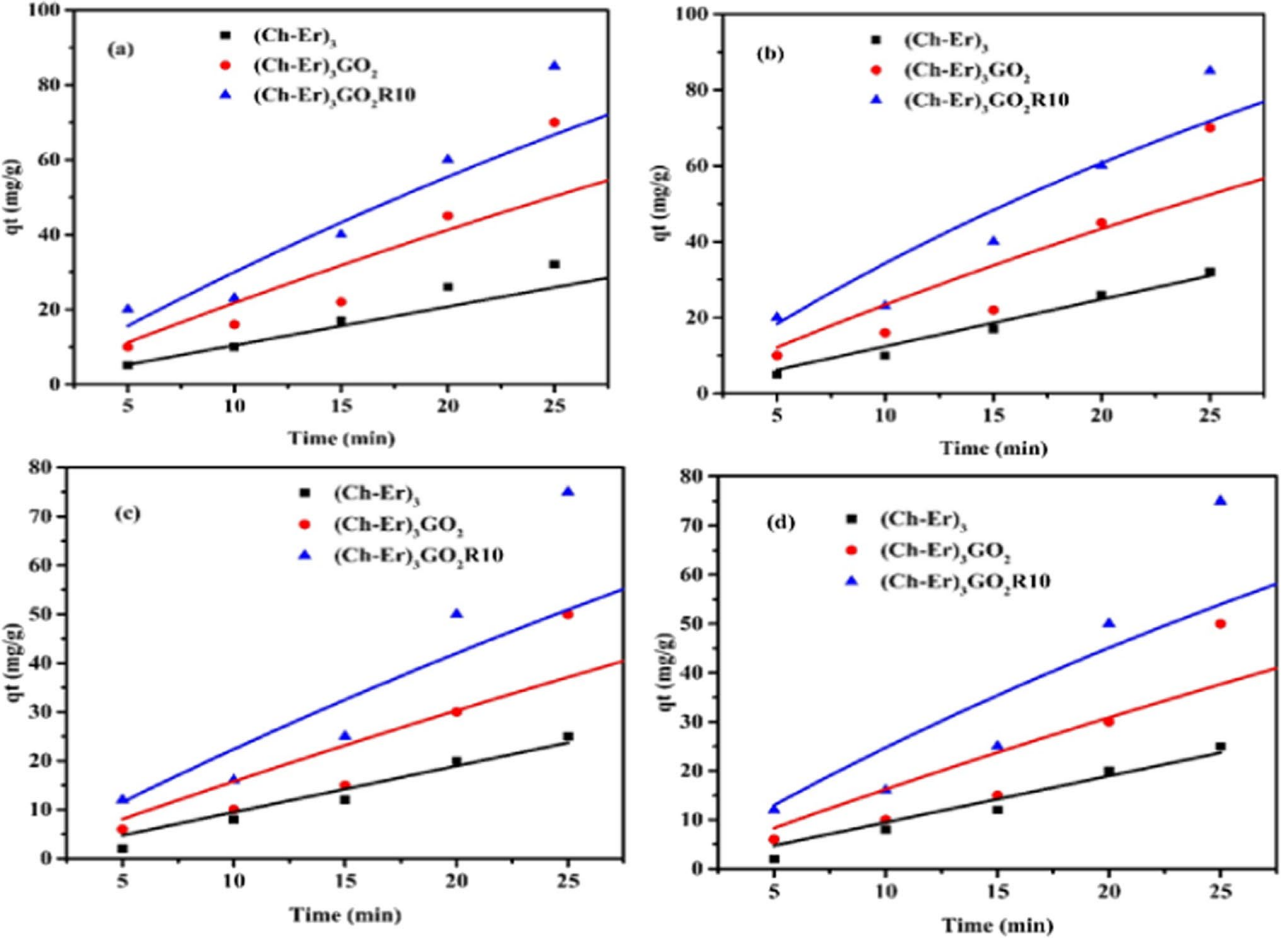
Kinetic adsorption curves for adsorption of MB dye (**a** and **b**) and Hg^2+^ (**c** and **d**) onto (Ch-Er)_3_ and (Ch-Er)_3_GO_2_ and (Ch-Er)_3_GO_2_R_10_; PFO (**a** and **c**); PSO (**b** and **d**); initial MB dye conc. and Hg^2+^ ions 50 ppm; at 25 °C

When determining the adsorption effectiveness of an adsorbent, the study of adsorption kinetics is of utmost importance. This may assist researchers in developing adsorption devices and studying and explaining the mechanisms of the adsorption process. Pseudo-first order and pseudo-second order were applied according to Eqs. (2) and (3), as shown in Fig. 7a–d for adsorption of MB and Hg^2+^ onto (Ch-Er)_3_, (Ch-Er)_3_GO_2_, (Ch-Er)_3_GO_2_R_10_. Table 3 provides a summary of the kinetic parameters.

**Table 3:** Kinetic parameters for the adsorption of MB dye and Hg^2+^ ions

MB dye				
Kinetic model	Kinetic parameters	(Ch-Er)_3_	(Ch-Er)_3_GO_2_	(Ch-Er)_3_GO_2_R_10_
Pseudo-first order PFO	*K*_1_ (min^−1^) *qe* (Cal.), (mg/g) *qe* (exp), (mg/g) *R*^2^	7.9 × 10^−4^ 329 156 0.992	0.011 210 174 0.996	0.016 200 186 0.998
Pseudo-second order PSO	*K*_2_ (g mg^−1^ min^−1^) *qe* (Cal.), (mg/g) *qe* (exp), (mg/g) *R*^2^	3.2 × 10^−11^ 496 156 0.993	2.7 × 10^−5^ 304 174 0.996	5.6 × 10^−5^ 264 186 0.998
Hg ^2+^				
Pseudo-first order PFO	*K*_1_ (min^−1^) *qe* (Cal.), (mg/g) *qe* (exp), (mg/g) *R*^2^	7.3 × 10^−5^ 130.06 156 0.98	0.0083 198.94 174 0.996	0.013 183.8 186 0.997
Pseudo-second order PSO	*K*_2_ (g mg^−1^ min^−1^) *qe* (Cal.), (mg/g) *qe* (exp), (mg/g) *R*^2^	5.4 × 10^−7^ 416.33 156 0.986	2.7 × 10^−5^ 304 174 0.994	5.6 × 10^−5^ 264 186 0.999

It can be deduced from the data that the values of the correlation coefficient, *R*^2^, associated with the pseudo-second-order model for both MB dye and Hg^2+^ are larger than those derived from the pseudo-first-order model. The *R*^2^ values, near 1, imply that the pseudo-second-order kinetic model explained the adsorption better than the pseudo-first-order kinetic model. This suggests that the adsorption process comprises chemisorption and that ion exchange between the sorbent and adsorbate was the rate-limiting step in the adsorption of MB dye and Hg^2+^ onto chitosan sorbents. This conclusion is consistent with a previous work that used the iodate-chitosan-constructed composite to remove MB dye (Wang and Zhuang 2017) and cross-linked chitosan-epichlorohydrin bio-beads to remove RR 120 dye (Jawad et al. 2019). The result shows that *K*_1_ and *K*_2_ values calculated from both the pseudo-first-order kinetic model and the pseudo-second-order kinetic model, respectively, were higher; (Ch-Er)_3_GO_2_R_10_ > (Ch-Er)_3_GO_2_ > (Ch-Er)_3_, indicating that the adsorption of MB dye and Hg^2+^ onto (Ch-Er)_3_GO_2_R_10_ is higher than (Ch-Er)_3_GO_2_ and much faster than (Ch-Er)_3_ and GO plays a significant role in the adsorption process.

#### Effect of initial pollutant concentration 

**Fig. 8:**
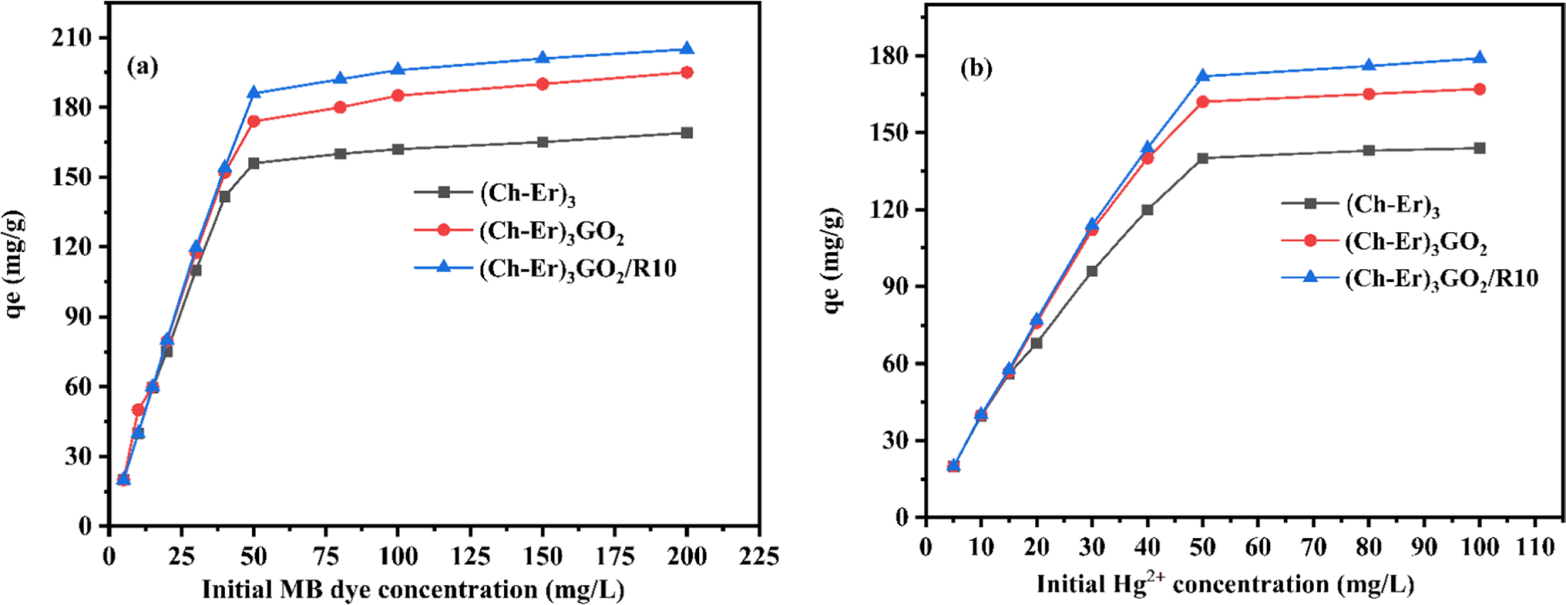
Effect of initial MB (**a**) and Hg ^2+^ (**b**) concentration on removal capacity (*qe* mg/g) of (Ch-Er)_3_, (Ch-Er)_3_GO_2_, and (Ch-Er)_3_GO_2_R_10_; adsorbate weight 0.1 g at room temperature

Using a series of various concentrations (0–200 mg L^−1^ for MB and 0–100 mg L^−1^ for Hg^2+^), the influence of the initial adsorbate concentration on the adsorption of MB dye onto (Ch-Er)_3_, (Ch-Er)_3_GO_2_, and (Ch-Er)_3_GO_2_R_10_ was examined. The adsorption capacities are illustrated in Fig. 8. Because all of the active sites accessible for adsorption were occupied, the clearance % of dye and metal cation dropped with increasing initial adsorbate concentration. However, because of the large driving force for mass transfer at a high starting dye concentration, the quantity of adsorbed species grows with time (Pama et al. 2019).

##### Adsorption isotherm models

**Fig. 9:**
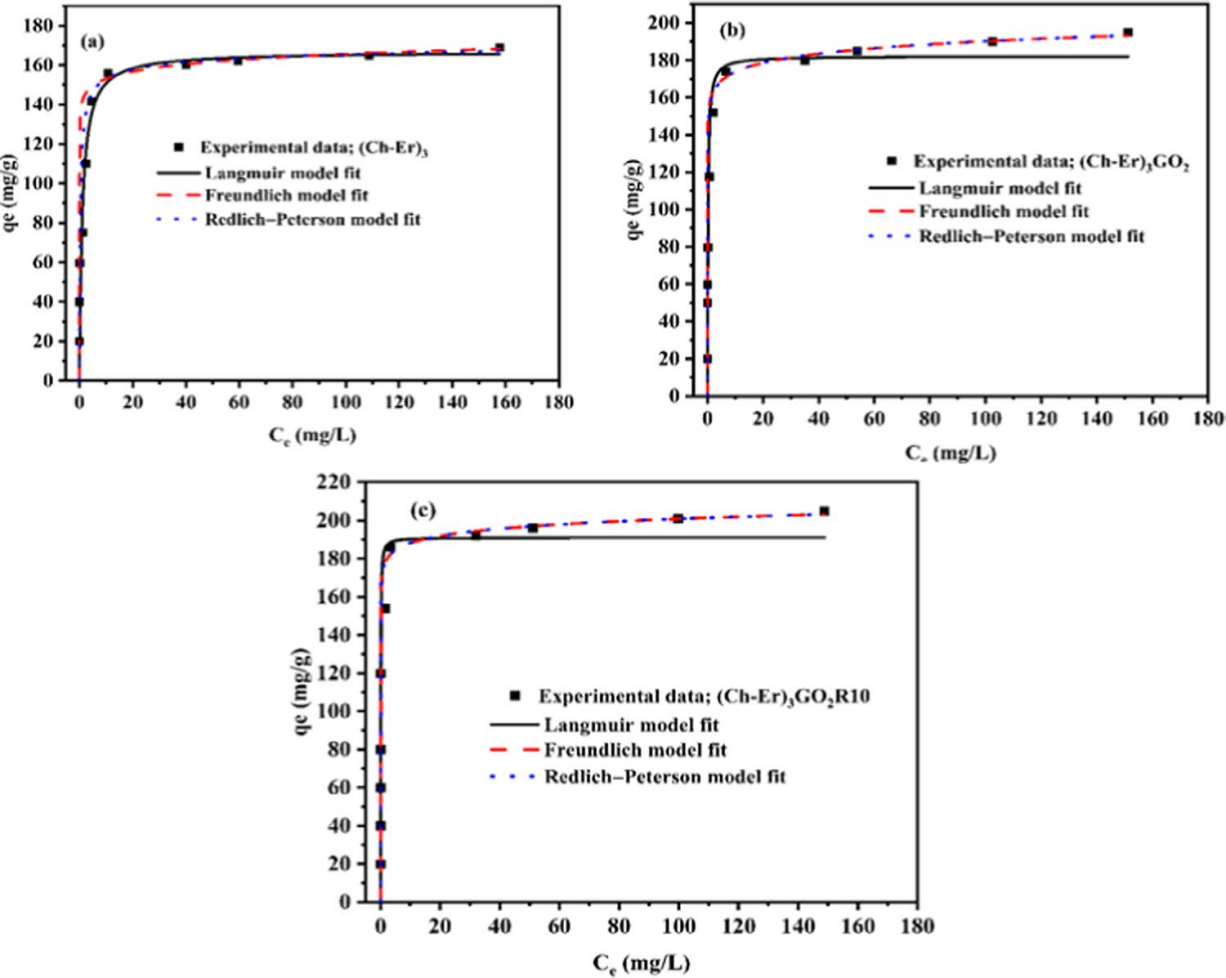
Adsorption isotherm models of MB dye onto (Ch-Er)_3_ (**a**); (Ch-Er)_3_GO_2_ (**b**) and (Ch-Er)_3_GO_2_R_10_ (**c**) at temperature 25 °C

**Fig. 10:**
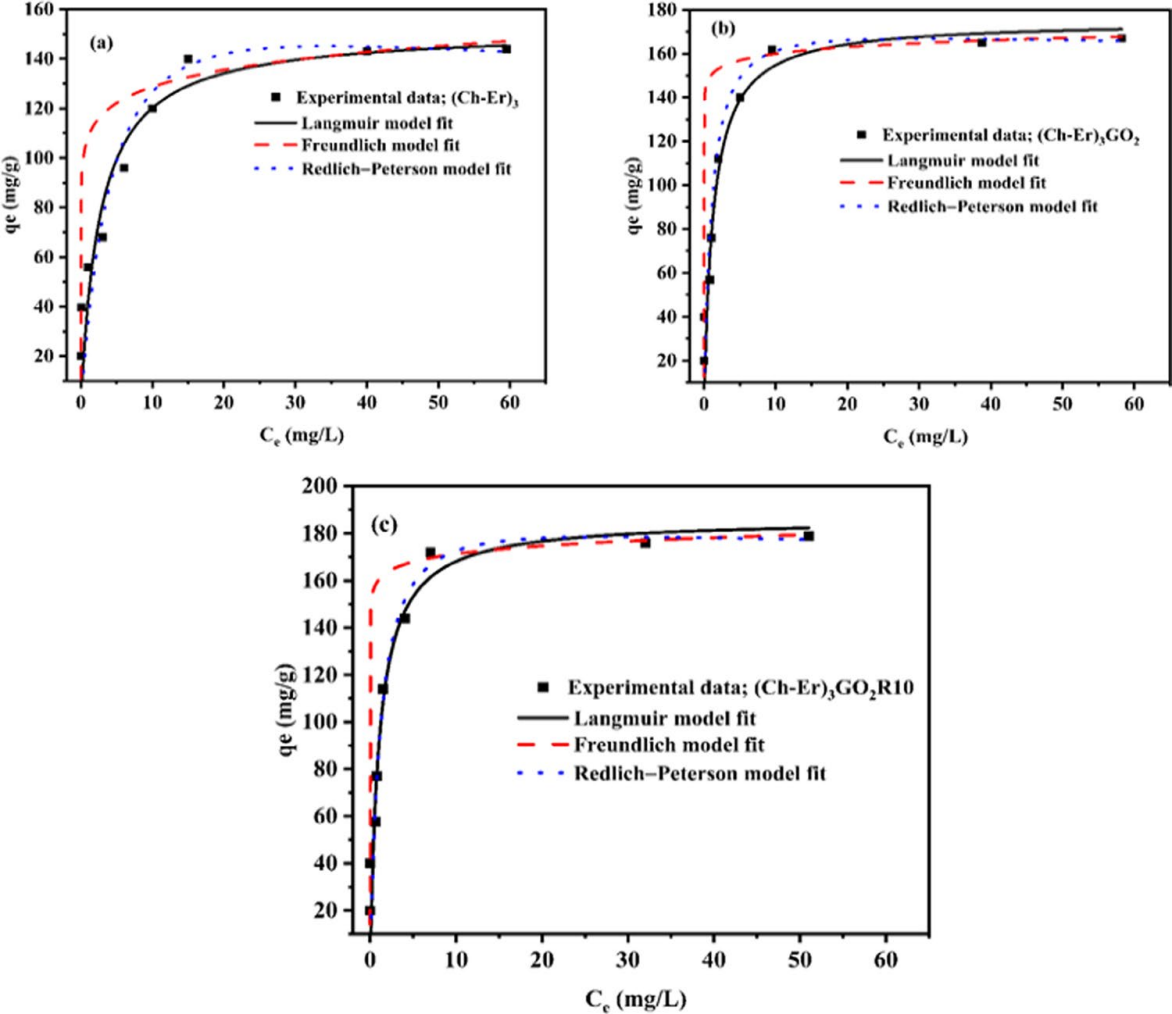
Adsorption isotherm models of Hg^2+^ onto (Ch-Er)_3_ (**a**); (Ch-Er)_3_GO_2_ (**b**) and (Ch-Er)_3_GO_2_R_10_ (**c**) at temperature 25 °C

Adsorption isotherms are critical for describing the nature of any system’s interaction between adsorbate and adsorbent. The affinity of the adsorbate for the sorbent may be deduced from the parameters derived from the connection between the concentration of the pollutant on the sorbent surface and the concentration of the pollutant in the bulk solution at a particular temperature. The characteristics obtained from various models provide crucial data on the adsorbent’s sorption processes, surface features, and affinities. There are a variety of formulae for interpreting experimental adsorption equilibrium data (Al-Ghouti and Da'ana 2020). In the present study, Langmuir, Freundlich, and Redlich-Peterson isotherms models were applied. All models were fit by engaging the non-linear fitting method by Origin Pro. 2016 software. The data are tabulated in Table 4. The theoretical isotherm model most fit is the one that describes the behavior of (Ch-Er)_3_, (Ch-Er)_3_GO_2_, and (Ch-Er)_3_GO_2_R_10_ towards the adsorption of MB dye and Hg^2+^. It was chosen from correlation coefficient (*R*^2^) values (Figs. 9 and 10). It can be seen that the *R*^2^ of the non-linear form of isotherm equations for tested dyes and heavy metal ions for the Redlich-Peterson model is substantially closer to unity than for the other two models, which confirms the complex nature of the removal process, which involves multistep adsorption on the surface and within the matrix. Finally, the favorability of (Ch-Er)_3_GO_2_R_10_ as an effective sorbent can be compared with other relevant sorbents (Table 5).

**Table 4:** Parameters of adsorption isotherm models of MB and Hg ^2+^

MB dye	Isotherm models	Parameter value	(Ch-Er)_3_	(Ch-Er)_3_GO_2_	(Ch-Er)_3_GO_2_R10
	Langmuir	*Q*_*m*_ (mg/g) *K*_*L*_ (L/mg) *R*^2^ *Q* exp. (mg/g)	166.7 0.94 0.8612 168.9	182.24 5.09 0.81244 195	191.1 21.91 0.73503 205
	Freundlich	*K*_*F*_ *n*_*F*_ *R*^2^	141.52 29.35 0.96613	158.84 25.5 0.95239	176.15 35.05 0.92576
	Redlich-Peterson	*A*_RP_ ${a}_{R}$ *g* *R*^2^	0.49 × 10^3^ 3.28 097866 0.99966	12.51 × 10^3^ 78.6 0.96144 0.99974	1.07 × 10^3^ 60.6 0.97148 0.99972
Hg ^2+^	Langmuir	*Q*_*m*_ (mg/g) *K*_*L*_ (L/mg) *R*^2^	152 0.379 0.87822	174 0.7676 0.91591	186.23 0.92829 0.92693
	Freundlich	*K*_*F*_ [(mg g^−1^)(mg^−1^)^1/n^] *n*_*F*_ *R*^2^	108.29 13.36 0.99439	150.21 36.77 0.9324	160.36 34.91 0.98736
	Redlich-Peterson	*A*_RP_ ${a}_{R}$ *g* *R*^2^	37.66 0.148 1.125 0.99929	156.27 0.80193 1.03518 0.99969	148.80 0.6888 1.044 0.93999

**Table 5:** The maximum adsorption capacity of obtained in the present study and other chitosan-based sorbents

	Adsorbent	Adsorption capacity	References
MB dye	Trimellitic anhydride isothiocyanate/carboxymethyl chitosan	434.8 mg g^−1^	[(Mohamed et al. 2021)]
	Graphene oxide–chitosan (GO–CS) hydrogel	300 mg g^−1^	[(Chen et al. 2013)]
	Chitosan/acrylic acid/TiO_2_	343 mg g^−1^	[(Mahmoud et al. 2020)]
	(Ch-Er)_3_ (Ch-Er)_3_GO_2_ (Ch-Er)_3_GO_2_R10	168.9 mg g^−1^ 195 mg g^−1^ 205 mg g^−1^	The present study
Hg^2+^	Chitosan sponge-like, chitosan flakes Chitosan sponge-like, chitosan foams	850 mg g^−1^ 350 mg g^−1^	[(Allouche et al. 2014)]
	Multi-cyanogunidine modified magnetic chitosan (CG-MCS nano-absorbent)	285 mg g^−1^	[(Wang et al. 2013)]
	Cross-linked magnetic chitosan-phenylthiourea (CSTU) resin	135–137 mg g^−1^	[(Monier and Abdel-Latif 2012)]
	(Ch-Er)_3_ (Ch-Er)_3_GO_2_ (Ch-Er)_3_GO_2_R10	152 mg g^−1^ 174 mg g^−1^ 186.23 mg g^−1^	The present study

#### Thermodynamic parameters of adsorption

The adsorption thermodynamics was conducted at 298, 308, and 318 K to explore the nature of the adsorption onto the modified adsorbents and to display the temperature impacts on MB and Hg^2+^ ions uptake. The thermodynamics of adsorptions was evaluated from the thermodynamic parameters, such as entropy change (∆*S*), enthalpy change (∆*H*), and Gibb’s free energy change (∆*G*), which can be calculated as follows:

7 $$\mathrm{ln}\frac{{q}_{e}}{{C}_{e}}=\frac{\Delta S}{R}-\frac{\Delta H}{RT}$$

8 $$\Delta G= \Delta H-T\Delta S$$

**Fig. 11:**
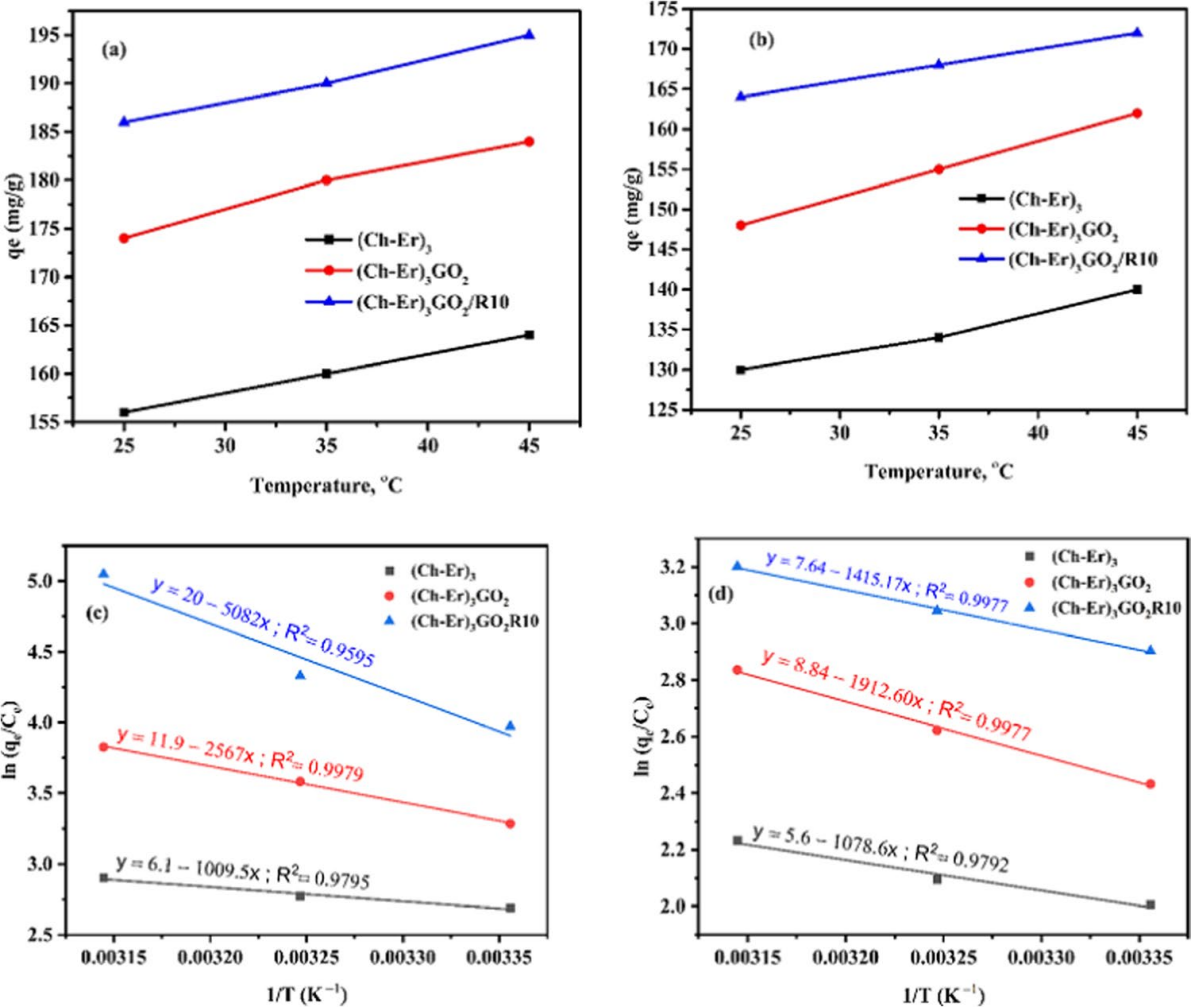
Effect of temperature on adsorption of MB dye (**a**) and Hg^2+^ (**b**) by (Ch-Er)_3_ and (Ch-Er)_3_GO_2_ and (Ch-Er)_3_GO_2_R10; Van’t Hoff plot for adsorption of MB dye (**c**) and Hg^2+^ (**d**) initial MB dye conc and Hg^2+^ ions. 50 mg/L; adsorpate weight 0.1 g

*R* is the universal gas constant (8.314 J mol^−1^ K^−1^), and *T* is the absolute temperature (K). The values of ∆*H* and ∆*S* can be obtained from the slope and intercept of the linear graph about $\mathrm{ln}\frac{{q}_{e}}{{C}_{e}}$ versus 1/*T*, as shown in Fig. 11, and the calculated parameters are listed in Table 6. It is recognized that the Gibbs free energy represents the degree of freedom of the sorption process, and a greater negative value implies more energetically advantageous sorption. The spontaneous nature of adsorption and the thermodynamic feasibility of the process is confirmed by negative values of $\Delta G$ (Guerrero-Fajardo et al. 2020).

**Table 6:** Thermodynamic parameters of MB dye and Hg^2+^ ions

MB dye				
Thermodynamic parameter		(Ch-Er)_3_	(Ch-Er)_3_GO_2_	(Ch-Er)_3_GO_2_R10
**∆***G* (kJ mol^−1^)	Temp. (K)			
	298 308 318	− 6.50 − 7.00 − 7.50	− 8.46 − 9.46 − 10.46	− 8.41 − 10.11 − 11.81
∆*S* (kJ mol^−1^ K^−1^)		0.05	0.10	0.17
∆*H* (kJ mol^−1^)		8.4	21.34	42.25
Hg^2+^ ions				
Thermodynamic parameter		(Ch-Er)_3_	(Ch-Er)_3_GO_2_	(Ch-Er)_3_GO_2_R10
∆*G* (kJ mol^−1^)	Temp. (K)			
	298 308 318	− 4.75 − 5.21 − 5.67	− 4.96 − 5.66 − 6.36	− 7.01 − 7.64 − 8.27
∆*S* (kJ mol^−1^ K^−1^)		0.046	0.07	0.063
∆*H* (kJ mol^−1^)		8.96	15.90	11.76

The positive value of $\Delta H$ indicated the endothermic nature of the adsorption; therefore, energy is required for the procedures to occur (Pholosi et al. 2020), while the positive values of $\Delta S$ reveal the increase in randomness for solid-solution interface and the value of $\Delta S$ increases in the order (Ch-Er)_3_GO_2_R_10_ > (Ch-Er)_3_GO_2_ > (Ch-Er)_3_ which proves a respectable affinity of (Ch-Er)_3_GO_2_R_10_ to absorb MB dye and Hg^2+^ (Babakhani and Sartaj 2020). All the results indicated that (Ch-Er)_3_GO_2_R_10_ was an efficient candidate for removing MB dye and Hg^2+^ ions from aqueous solutions.

## Conclusion

The current study investigates the preparation and characterization of chitosan-modified products for simultaneous removal of MB and Hg^2+^ from simulated solution. Several factors were verified:

- The effect of chitosan/erythritol ratio: Four ratios of chitosan/erythritol were applied, and the products were denoted as (Ch-Er)_1_, (Ch-Er)_2_, (Ch-Er)_3_, and (Ch-Er)_4_. They were investigated via an AFM for their topography and surface properties. A quick preliminary test was accomplished to investigate their removal performance towards methylene blue dye and mercury cation from a simulated solution. The data revealed that the optimized sample was (Ch-Er)_3_GO_2_.
- The effect of graphene oxide ratio: The optimized sample was further modified by different graphene oxide content. The removal experiments verified an optimum graphene oxide ratio above which the removal capacity decreased remarkably, and the optimum sample was (Ch-Er)_3_GO_2_.
- The effect of irradiation dose: The surface of the optimum sample was further modified with two electron beam irradiation doses, 10 and 20 kGy. The data revealed that the increase in irradiation dose caused clustered cross-linking regions to form, which altered the surface properties and decreased the removal performance. All the modifications mentioned above were proved by the AFM.
- The effect of initial pollutant concentration on the removal performance of (Ch-Er)_3_, (Ch-Er)_3_GO_2_, and (Ch-Er)_3_GO_2_R_10_ was studied, and it was found that the values of *q*_*e*_ increase by increasing the concentration of the contaminant and the rate of removal decreases.
- The kinetic study was conducted at pseudo-first-order, pseudo-second-order kinetic models for the adsorption of MB and Hg^2+^ onto (Ch-Er)_3_, (Ch-Er)_3_GO_2_, and (Ch-Er)_3_GO_2_R_10_. The investigated samples followed pseudo-second order, which implied a chemisorption process.
- Three adsorption isotherm models were applied to verify the sorption mechanism: Langmuir, Freundlich, and Redlich-Peterson. The data demonstrated that the most fitted model is Redlich-Peterson, revealing the complex nature of the removal process.
- The thermodynamic parameters of adsorption showed that the process is endothermic.
- A comparison was held between the maximum removal capacity of (Ch-Er)_3_GO_2_R_10_ and other chitosan-based sorbents, and it was shown that (Ch-Er)_3_GO_2_R_10_ is an excellent sorbent material for both MB dye and Hg^2+^ ions.

## Supplementary Information

Below is the link to the electronic supplementary material.

Supplementary file1 (DOCX 2672 KB)

## Data Availability

The data supporting this study’s findings are available in the supplementary materials.
