# Supplementary material for: Green synthesis of chitosan/erythritol/graphene oxide composites for simultaneous removal of some toxic species from simulated solution
Source: Environ Sci Pollut Res Int. 2022 Nov 9;30(10):25903–19. doi: 10.1007/s11356-022-23951-4 (PMC9995588; doi:10.1007/s11356-022-23951-4)
Supplement: Supplementary file 1 — Supplementary file1 (DOCX 2672 KB) [file 11356_2022_23951_MOESM1_ESM.docx]

**Supplementary Data**

**Green synthesis of Chitosan/Erythritol/Graphene oxide composites for simultaneous removal of some toxic species from simulated solution**

Asma Sayed^1*^, Azza M. Mazrouaa^2^, Manal G. Mohamed^2^, Manar El-Sayed Abdel-Raouf^3^

^1^Polymer Chemistry Department, National Center for Radiation Research and Technology, Egyptian Atomic Energy Authority, Nasr City, Cairo, Egypt

^2^ Polymer Lab, Department of Petrochemicals, Egyptian Petroleum Research Institute, Nasr City, Cairo, Egypt

^3^Additives Lab, Department of Petroleum Application, Egyptian Petroleum Research Institute, Nasr City, Cairo, Egypt

* Corresponding author e-mail: [asmaasayedncrrt@gmail.com](mailto:asmaasayedncrrt@gmail.com)

**Table of Content:**

| **1-** | **Method of Preparation of Chitosan/ Meso-Erythritol hydrogel** |
| --- | --- |
| **2** | **Chemical modification of chitosan** |
| **3-** | Figure S1: Schematic representation of synthetic procedure of chitosan/erythritol |
| **4-** | Figure S2: FTIR spectrum of Ch, Er, and (Ch-Er)_3_GO_2_ |
| **5-** | Figure S3: AFM images of chitosan surface, (Ch-Er)_1_ ,(Ch-Er)_2_, (Ch-Er)_3_ and (Ch-Er)_4_ |
| **6-** | Fig.S4 SEM of Ch pure, (Ch-Er)_3_ and (Ch-Er)_3_GO_2_ |
| **7-** | Fig.5S Removal performance of the (Ch-Er) series towards MB (a) and Hg ^2+^ (b) (50ppm, pH7 and at 25 ^o^C). |
| **8-** | Fig. S6 Removal capacity (qe mg/g) at different time intervals (min) of MB (a) and Hg2+ (b); initial conc.50 ppm; adsorbate weight 0.1g at room temperature; (Ch-Er)3, (Ch-Er)3GO2 and (Ch-Er)3GO2R10 |
| **10-** | Table S1: The most important peaks |
| **11-** | Table S2: AFM data of (Ch-Er)_1_ ,(Ch-Er)_2_, (Ch-Er)_3_ and (Ch-Er)_4_ |
| **12-** | References |

1. **Method of preparation of Chitosan/ Meso-Erythritol hydrogel**

Modified chitosan films were prepared by dispersing 50 g chitosan powder in 500 mL of acidified water (2% acetic acid) while stirring on a magnetic stirrer until complete solubility. The solution was filtered to remove undissolved impurities using vacuum filter. After filtration, the solution was returned back to the sonicator, then meso-erythritol (Er) (different ratio 10%, 20%, 30% and 40% Er to Ch) was added and the solution and sonicated again to achieve complete dispersion. The solutions were poured into clean petri dishes and allowed to dry in a well- ventilated oven at 60 °C for approximately 1 day. The formulations were coded according to the percentage of erythritol as (Ch-Er)_1_, (Ch-Er)_2_, (Ch-Er)_3_ and (Ch-Er)_4_.

**2. Chemical modification of chitosan:**

Chitosan is a highly functional green carbohydrate biopolymer having hydroxyl groups and highly reactive amino groups ([Hsan et al. 2019](#_ENREF_2)) that can be adapted by different chemical procedures to fit various applications. The applications of chitosan are limited due to its poor solubility in water and inferior mechanical properties. The chemical modifications for functionalizing the chitosan structure are the N-substitution, O-substitution (with or without protecting the reactive sites of the chitosan) and cross-linking with other compounds; these chemical modifications allow improvement of its chemical and physical properties ([Zhao et al. 2016](#_ENREF_3)). Chitosan is obtained by deacetylation of chitin. The presence of amino functional groups in chitosan increases its adsorption capacity compared to its precursor and characterized by its degree of deacetylation. In the present work, the degree of deacetylation is >75% which means the presence of numerous amine groups ready for chemical reaction. Combination of chitosan with sugars can be performed by Millard reaction ([Corazzari et al. 2015](#_ENREF_1)). Direct dehydration reaction involves the interaction of the -OH of erythritol and -NH_2_ groups of chitosan to form a network structure which is then supported by incorporation of graphene oxide powder within the network. The product with new properties allows the chitosan to be used in water engineering (bioadsorbence of heavy metals and dyes). The mechanism of the prepared modified chitosan is shown in Figure S1


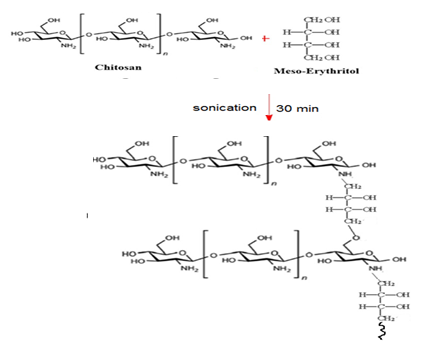


Figure S1: Schematic representation of synthetic procedure of chitosan/erythritol

**
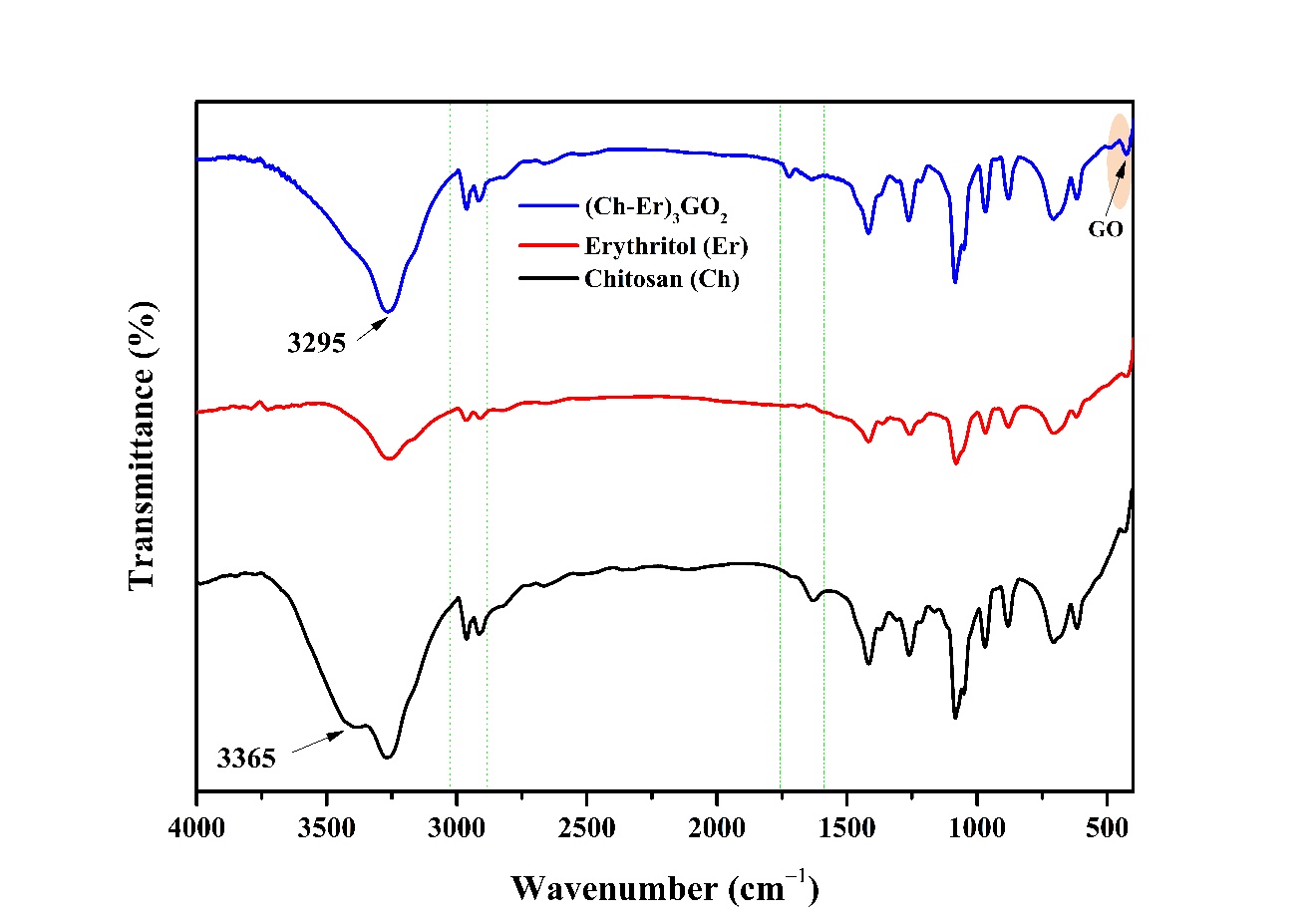
**

Figure S2: FTIR spectrum of Ch, Er, and (Ch-Er)_3_GO_2_

| 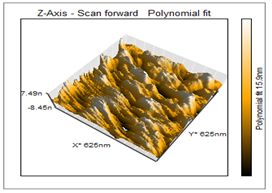  AFM image of chitosan surface | 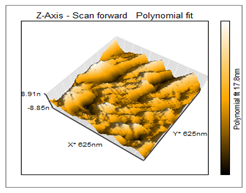  AFM image of (Ch-Er)_1_ |
| --- | --- |
| 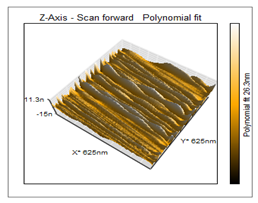  AFM image of (Ch-Er)_2_ | 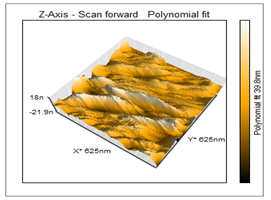  AFM image of (Ch-Er)_3_ |
| 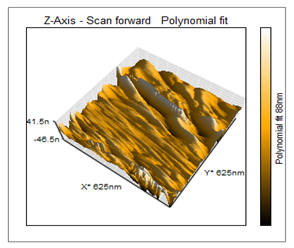  AFM image of (Ch-Er)_4_ | |

Figure S3: AFM images of chitosan surface, (Ch-Er)_1_ ,(Ch-Er)_2_, (Ch-Er)_3_ and (Ch-Er)_4_


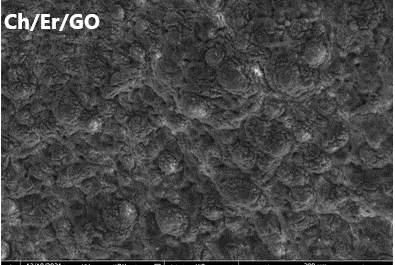

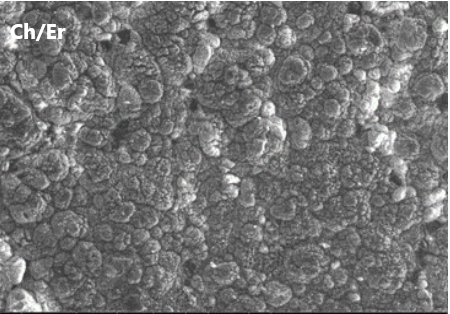

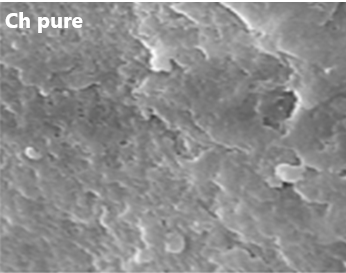


Fig.S4 SEM of Ch pure, (Ch-Er)_3_ and (Ch-Er)_3_GO_2_

**
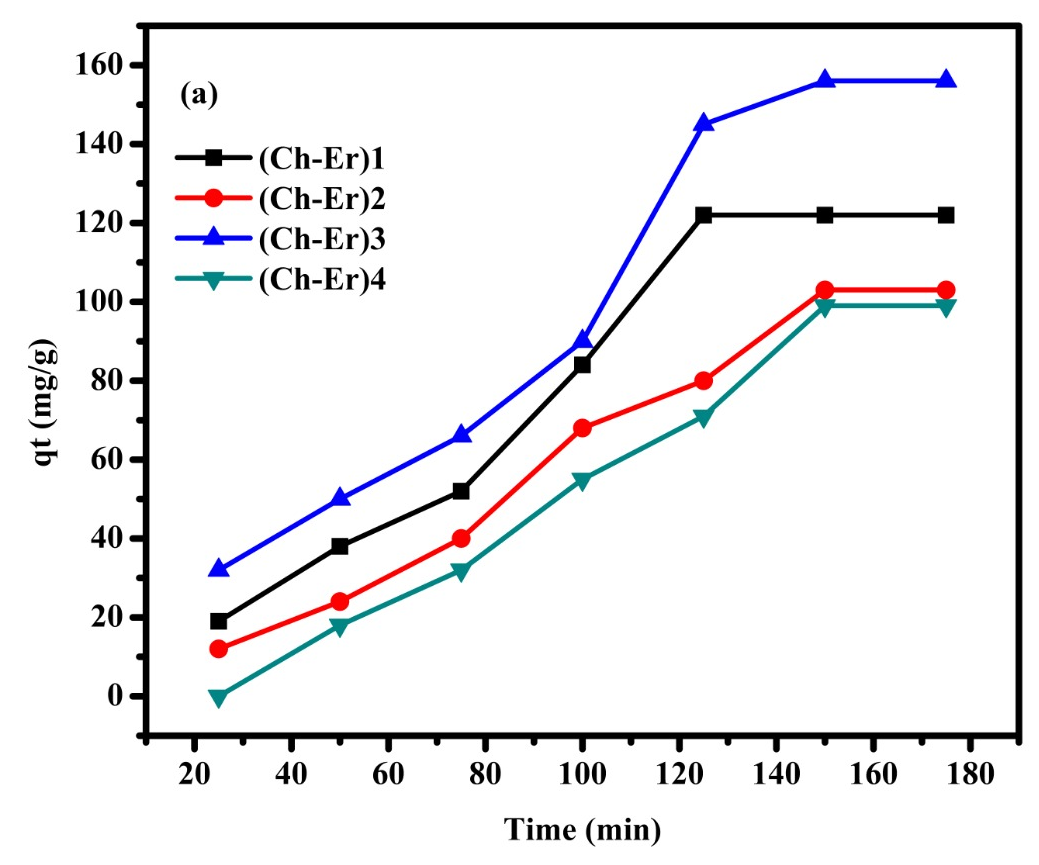

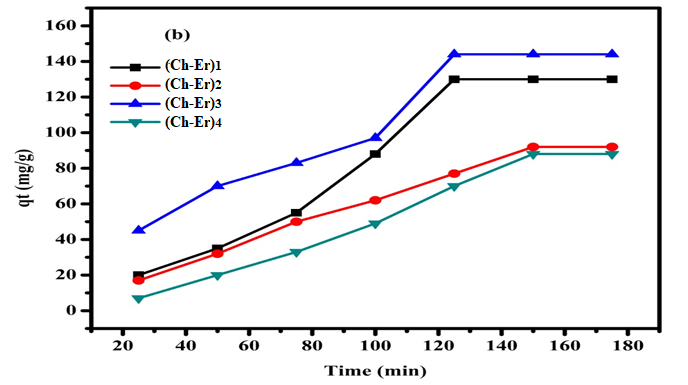
**

Fig.5S Removal performance of the (Ch-Er) series towards MB (a) and Hg ^2+^ (b) (50ppm, pH7 and at 25 ^o^C)

| 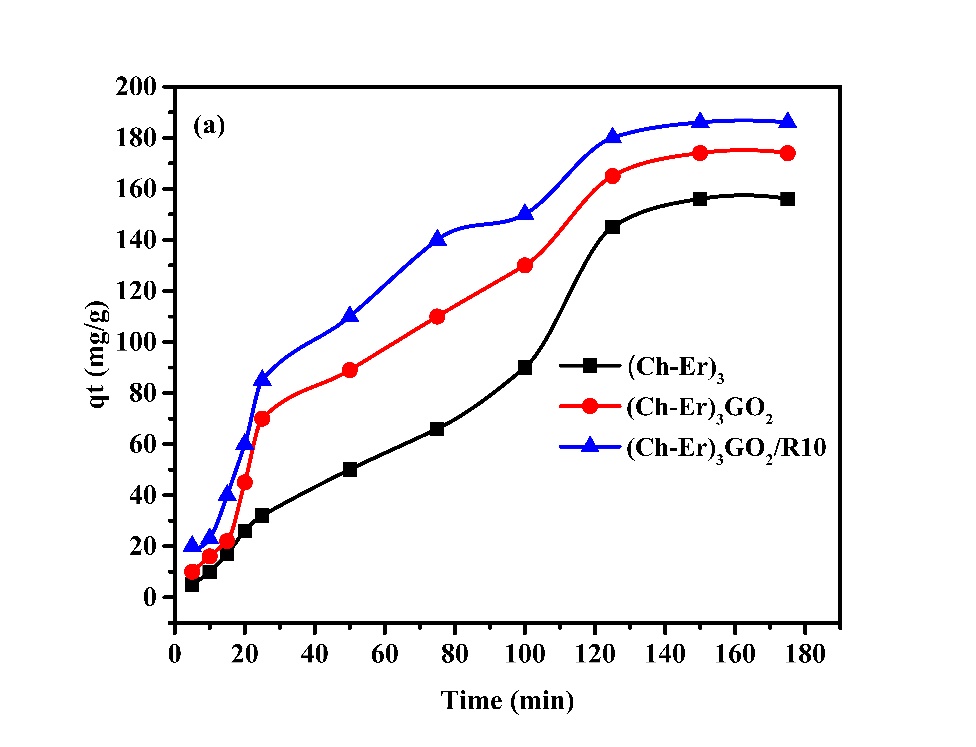 | 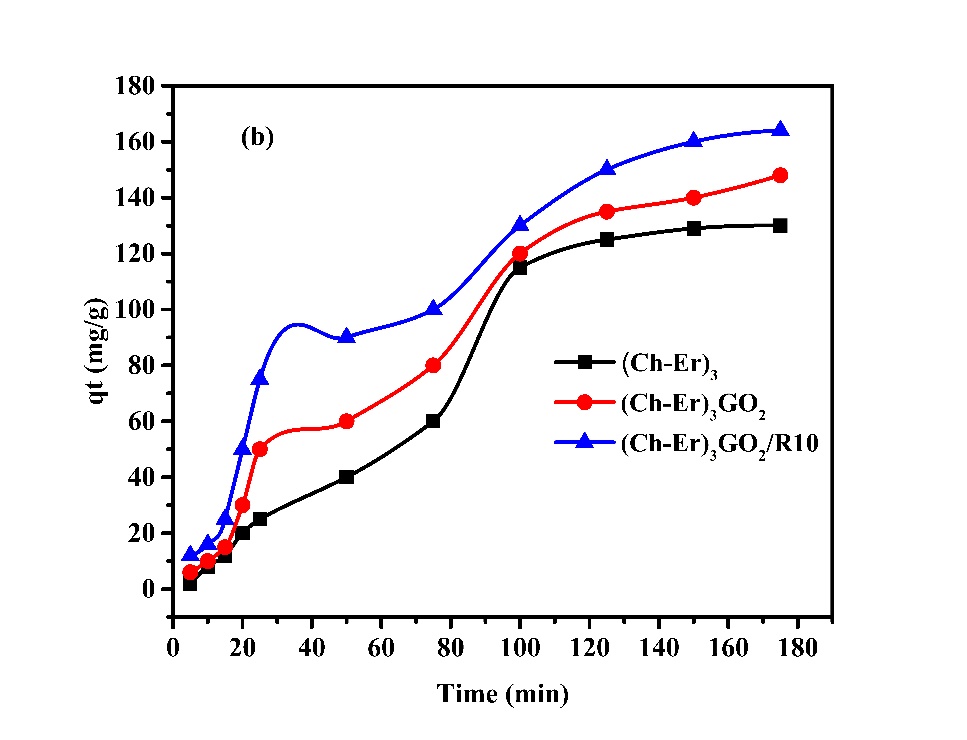 | |
| --- | --- | --- |
| Fig. S6 Removal capacity (qe mg/g) at different time intervals (min) of MB (a) and Hg^2+^ (b); initial conc.50 ppm; adsorbate weight 0.1g at room temperature; (Ch-Er)_3_, (Ch-Er)_3_GO_2_ and (Ch-Er)_3_GO_2_R10 | | |

Table S2: The most important peaks

| The compound | The peaks | The interpretation |
| --- | --- | --- |
| Chitosan | Strong peaks at 1033 cm^–1^ and 1061 cm^−1^  Medium peak at 2890 cm^–1^  A strong band in the region 3295–3365 cm^−1^.  Absorption bands at around 2882 and 2877 cm^−1^  Two overlapped bands at 1590 and 1640 cm^−1^  Strong bands at around 1375. | C–O–C stretching  CH_2_ stretching  N-H and O-H stretching.  C-H symmetric and asymmetric stretching.  N-H bending of amide  CH_2_ bending |
| Erythritol | strong peak at 3251 cm^−1^.  Two medium peaks at 2968 and 2957 cm^−1^.  Absorption band at 1181 cm^−1^.  Significant peaks at 1560 and 1467 cm^−1^ | Stretching vibration of free O-H  C-H symmetric and asymmetric stretching  Asymmetric stretching of the C-O bond  CH_2_ bending and CH symmetrical |
| (Ch-Er)_3_GO_2_ | Broadened peak at 3280 cm^−1^  Peak at 1020 cm^-1^  Peaks at 435-520 cm^-1^ | O-H overlapped with N-H  C-N stretch  Characteristic peaks of GO |

Table S3: AFM data of Some Samples

| No. | Sample | Height | Scale | Data analysis | R_a_  _(Average area roughness)_ |
| --- | --- | --- | --- | --- | --- |
|  | Chitosan | 15.9nm | 625x625nm | Polynomial fit | 9.921nm |
|  | (Ch-Er)_1_ | 17.8nm | 625x625nm | Polynomial fit | 23.23nm |
|  | (Ch-Er)_2_ | 26.3nm | 625x625nm | Polynomial fit | 23.44nm |
|  | (Ch-Er)_3_ | 39.8nm | 625x625nm | Polynomial fit | 24.11nm |
|  | (Ch-Er)_4_ | 88nm | 625x625nm | Polynomial fit | 24.34nm |

References

Corazzari I, Nisticò R, Turci F, Faga MG, Franzoso F, Tabasso S, Magnacca G (2015): Advanced physico-chemical characterization of chitosan by means of TGA coupled on-line with FTIR and GCMS: Thermal degradation and water adsorption capacity. Polymer Degradation and Stability 112, 1-9

Hsan N, Dutta P, Kumar S, Bera R, Das N (2019): Chitosan grafted graphene oxide aerogel: Synthesis, characterization and carbon dioxide capture study. International journal of biological macromolecules 125, 300-306

Zhao D, Gao X, Wu C, Xie R, Feng S, Chen C (2016): Facile preparation of amino functionalized graphene oxide decorated with Fe3O4 nanoparticles for the adsorption of Cr (VI). Applied Surface Science 384, 1-9
